# Diversity and selected functional traits of microbiota associated with traditional dried plant foods from South African informal markets

**DOI:** 10.1093/femsmc/xtag026

**Published:** 2026-05-13

**Authors:** Birgit Wassermann, Isabella Kögl, Jarishma K Gokul, Wisnu Adi Wicaksono, Matthias Schweitzer, Lise Korsten, Gabriele Berg

**Affiliations:** Institute of Environmental Biotechnology, Graz University of Technology, Petersgasse 12, Graz 8010, Austria; Institute of Environmental Biotechnology, Graz University of Technology, Petersgasse 12, Graz 8010, Austria; Department of Plant and Soil Sciences, University of Pretoria, Private Bag X20, Hatfield 0028, South Africa; Centre for Microbial Ecology and Genomics, Department of Biochemistry, Genetics and Microbiology, University of Pretoria, Hatfield 0028, South Africa; Institute of Environmental Biotechnology, Graz University of Technology, Petersgasse 12, Graz 8010, Austria; Institute of Environmental Biotechnology, Graz University of Technology, Petersgasse 12, Graz 8010, Austria; Department of Plant and Soil Sciences, University of Pretoria, Private Bag X20, Hatfield 0028, South Africa; Department of Science and Innovation-National Research Foundation Centre of Excellence in Food Security, Pretoria, Private Bag X20, Hatfield 0028, South Africa; Institute of Environmental Biotechnology, Graz University of Technology, Petersgasse 12, Graz 8010, Austria; Leibniz Institute for Agricultural Engineering and Bioeconomy, Max-Eyth-Allee 100, Potsdam 14469, Germany; Institute for Biochemistry and Biology, University of Potsdam, Karl-Liebknecht-Str. 24-25, Potsdam OT Golm 14476, Germany

**Keywords:** edible microbiome, traditional food, food safety, baobab, marula, leafy greens

## Abstract

Traditional plant-based products provide nutritional benefits and support cultural heritage; however, their sale in urban informal markets raises potential food safety considerations. We characterized the microbiota of five traditional dried plant products (baobab, masau, nyii, dinawa, and lude) obtained from three informal markets in South Africa (*n* = 51 samples) using 16S rRNA gene sequencing and quantitative real-time PCR; bacterial isolates (*n* = 87) were further evaluated using selected phenotypic assays. Bacterial abundance and composition varied across products and vendors. Baobab exhibited the highest microbial richness (1460 ASVs) but relatively low bacterial loads (10^6^ 16S rRNA gene copies g^−1^), whereas dried leafy greens showed the lowest richness (470 ASVs) but the highest bacterial abundance (10^9^ copies g^−1^). Across products, higher bacterial diversity correlated with genera such as *Bifidobacterium* and *Prevotella*, while higher bacterial abundance correlated with genera such as *Salmonella, Vibrio*, and *Acinetobacter*. Notably, health implications of detected taxa cannot be inferred from genus-level identification based on 16S rRNA gene sequencing. Phenotypic traits observed among selected isolates included growth in the presence of several antibiotics (particularly sulfadiazine and ampicillin), protease activity, and inhibition of indicator strains under laboratory conditions. Overall, traditional dried plant foods harbor diverse microbial communities shaped by plant characteristics and vendor-related practices, highlighting the importance of improved handling and drying practices.

## Teaser

Traditional dried plant foods host diverse microbes shaped by plant type and handling practices, highlighting the importance of drying and drying hygiene in urban informal markets.

## Introduction

The importance of traditional foods for nutrition and food security encompasses various cultural, social, and health aspects. Traditional foods are deeply connected to the cultural heritage and identity of communities, are crucial for income generation, and can serve as a buffer against food shortages (Wu et al. 2018, Zabel et al. 2019, VanBuren et al. 2020, Mahoney et al. 2022, Sparling et al. 2022). Moreover, the consumption of traditional and indigenous foods is closely related to the concept of food sovereignty (Van Der Ploeg 2014) and the knowledge to produce those foods can assist in creating sustainable food systems that respect local ecosystems and cultural practices (Schwan et al. 2007, Antonelli 2023). Reflecting their significance, regulatory frameworks such as the South African food-based dietary guidelines emphasize the inclusion of traditional foods in daily diets to support health and well-being (Du Plooy et al. 2018 ).

One aspect increasingly investigated in relation to traditional diets is their association with gut microbiome composition (De Filippo et al. 2010, Yatsunenko et al. 2012, Xu et al. 2015). Traditional foods have been associated with distinct and diverse gut microbiota profiles that support improved nutrient absorption and metabolic health when compared to industrialized diets (Schnorr et al. 2014, Obregon-Tito et al. 2015, Michalak et al. 2020). Beyond their nutritional value, fruits and vegetables harbor a diverse range of microorganisms on their surfaces and within their tissues (Leff and Fierer 2013, Berg et al. 2015, Wassermann et al. 2017, 2019, Wicaksono et al. 2022). Previous studies have reported functional traits of those bacteria, including metabolic activity, vitamin biosynthesis, production of short-chain fatty acids, or antagonistic activity toward pathogens, with such evidence derived either from in silico analyses, including metagenome-based meta-analyses, or from functional assessments conducted under controlled laboratory conditions (Soto-Giron et al. 2021, Serrano and Bezrutcyzk 2023, Wicaksono et al. 2023a, Carlino et al. 2024, Berg et al. 2025). Importantly, edible plants may also contain environmental or opportunistic microorganisms that are relevant to food safety, particularly when foods are processed, stored, or sold under conditions with limited hygiene infrastructure (Silva et al. 2017, Balali et al. 2020, Asfaw et al. 2022).

Urban informal markets in developing countries are often characterized by poor infrastructure, inadequate hygiene practices, and close proximity of food products to live animals, which may facilitate cross-contamination and the presence of microorganisms associated with zoonotic or antimicrobial-resistant contexts (Moyane et al. 2013, Naguib et al. 2021, Richter et al. 2021, Zavala Nacul and Revoredo-Giha 2022). Vendors are frequently unlicensed, lack formal food safety training, and operate without access to refrigeration or clean water (Grace 2023). To address some of these challenges, drying is commonly employed as a preservation strategy. Drying is one of the oldest known food preservation techniques, dating back to around 12 000 BCE in the Middle East. Since then, it has played a vital role in extending shelf life, preserving food, and reducing post-harvest losses (Santos et al. 2025). In South Africa, a variety of dried plant products are produced by local communities and sold at informal markets. Despite the cultural and nutritional importance of these foods, systematic characterization of their microbial communities, particularly those sold through informal market systems, has not yet been reported.

In this study, we focused on five such products: the leafy greens *Vigna unguiculata* (L.) WALP. (cowpea; “dinawa”) and *Cleome gynandra*  L. (spider plant; “lude”), collectively referred to as morogo, a term used to describe traditional leafy wild greens that are commonly foraged or cultivated and incorporated into local diets. In addition, the fruits *Ziziphus mauritiana* LAM. (“masau”) and *Berchemia discolor* (KLOTZSCH) HEMSL. (“nyii”), and the fruit pulp of *Adansonia digitate*  L. (“baobab”) were investigated. All plants, except for masau, which originated in the Indo-Malaysian region of South and Southeast Asia, are native to South Africa and are recognized for their nutritional value and ethnobotanical relevance in regional contexts (Takaidza 2024). All products are commonly consumed due to their nutrient content, including fiber, vitamins, and micronutrients, and are traditionally used in medicinal practices (Nyanga et al. 2013, Maroyi and Cheikhyoussef 2015, Takaidza 2024, The Editors of Encyclopaedia Britannica 2024). Baobab, in particular, which is one of the most iconic plant species in Africa, is widely recognized for its nutritional composition, including high vitamin C, antioxidant levels, and dietary fiber content (Garvey et al. 2017, Foltz et al. 2021, Duysburgh et al. 2024).

In the present study, we aimed to (i) characterize the bacterial community structure associated with five traditional dried plant foods sold in urban informal markets using 16S rRNA amplicon sequencing and to quantify microbial loads via qPCR; (ii) assess whether microbial composition varies according to product type, vendor, or market location; and (iii) screen selected isolates for phenotypic traits, including enzymatic activity, growth in the presence of selected antibiotics, and antagonistic activity towards indicator pathogens under laboratory conditions. Through this approach, the study provides baseline data on microbial diversity associated with traditional dried plant foods sold in informal markets.

## Material and methods

### Sample collection

**Figure 1:**
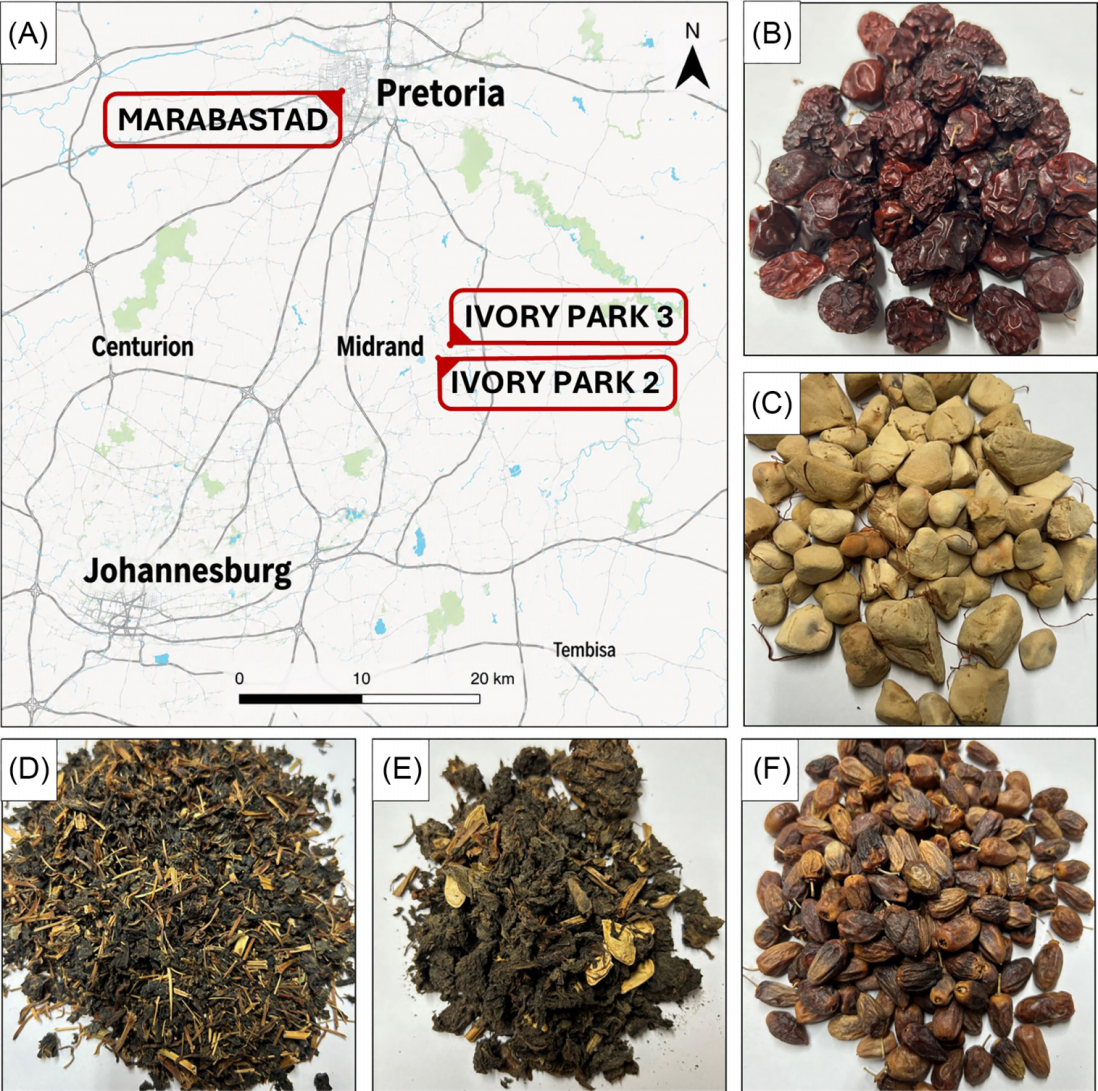
Locations of the three urban informal markets in Pretoria and Johannesburg (A), where the following types of traditional dried foods were obtained: masau (B), baobab (C), dinawa (D), lude (E), and nyii (F). Photographs were taken after purchase and transport to the laboratory. Map tiles by CARTO, data by OpenStreetMap, under ODbL. Photo credit: Jarishma K. Gokul.

The South African traditional dried plant products analyzed are produced from the fruits *Z. mauritiana* (“masau”) and *B. discolor* (“nyii”), the fruit pulp of *A. digitate* (“baobab”), and the leafy green vegetables *V. unguiculata* (cowpea, “dinawa”) and *C. gynandra* (spider plant, “lude’). The dried products were obtained from informal markets in the areas Marabastad (Pretoria, South Africa), Ivory Park 2, and Ivory Park 3 (City of Johannesburg Metropolitan Municipality, South Africa) between August and September of 2022. A convenience sampling strategy was applied, whereby all available vendors selling the respective target products at the time of visit were included. Samples were purchased in three biological replicates in plastic or paper bags from two to four vendors at each market. Masau and nyii were available from four vendors each, while baobab and lude were offered by three vendors. Dinawa was exclusively accessible from two vendors at Ivory Park 2 and Ivory Park 3, whereas it was not offered at Marabastad. The limited number of vendors available for some of the products reduces the statistical power to detect vendor-related effects, which should be considered when interpreting the results. In total, 51 food samples were obtained. Fruits and leafy greens were presented open-layered in bags or bowls. The dried powder of the baobab fruit pulp was offered in small containers. Figure 1 shows the sampling locations and images of dried products. Samples were stored in the original bags or containers under dry conditions at room temperature until further processing.

### Sample processing and DNA extraction

Prior to DNA extraction, dried dinawa and lude leaves were crushed with a mortar and pestle. The dried fruits, nyii and masau, were first soaked in sterile 0.85% NaCl overnight to allow the removal of the seeds. This was done to ensure that only the microbial communities associated with the edible pulp, rather than those within the seeds, were included in the analysis. Afterwards, the fruit peel and pulp were crushed with a mortar and pestle. For baobab, the pulp was ground from the seeds with a mortar and pestle. After sieving, baobab powder was soaked in sodium phosphate buffer (5%; component of the FastDNA® SPIN Kits, MP Biomedicals, Santa Ana, CA, USA) until saturation, to prevent the absorption of buffers during subsequent DNA extraction. The total community DNA was extracted from 0.3 g of each sample in three technical replicates using the FastDNA Spin Kit for Soil (MP Biomedicals, Santa Ana, CA, USA), according to the manufacturer’s protocol. Extracting DNA from baobab samples presented significant challenges, likely due to their elevated protein content (Chadare et al. 2008). We successfully implemented the following protocol, which was subsequently applied to all other samples to maintain consistency in the DNA extraction process. Post DNA extraction with the FastDNA Spin Kit for Soil, samples were additionally purified using the MasterPure Complete DNA & RNA Purification Kit (Epicentre, Madison, WI, USA) with the following modifications. After adding 400 µL of 2× T&C solution (Epicentre, Madison, WI, USA) and 400 µL of MPC solution (Epicentre, Madison, WI, USA) to the extracted DNA, cell debris was pelleted by centrifugation at 4°C for 10 min at 14 000 *g*. The supernatant was transferred to a clean microcentrifuge tube. An amount of 200 µL of 10% polyvinylpyrrolidon (PVP) solution (Merck, Darmstadt, Germany) was added, and the mixture was vortexed and centrifuged at 4°C for 10 min at 14 000 *g*. To precipitate the DNA, the supernatant was mixed with 500 µL of isopropanol and incubated on ice for 5 min. The suspension was centrifuged at 4°C for 10 min at 14 000 *g*, and the supernatant was discarded. The resulting DNA pellet was rinsed twice with 100 µL of 80% ethanol. The washed DNA pellet was resuspended in 50 µL of TE buffer (10 mM Tris-HCl [pH 8.0], 1 mM EDTA, Epicentre, Madison, WI, USA) and stored at −20°C until further processing.

### Quantification of bacteria in traditional dried foods

Absolute bacterial abundance in the traditional dried products was determined by quantitative real-time PCR (qPCR) based on SYBR Green fluorescence using the qTOWER³/G (Analytik Jena, Jena, Germany). The variable regions V4 of the 16S rRNA of bacteria were amplified using the universal primer pair 515f–806r (Caporaso et al. 2011) and the following cycling conditions: initial denaturation at 95°C, followed by 40 cycles of denaturation at 95°C for 5 s, annealing at 54°C for 20 s, PNA step at 78°C for 5 s, extension at 72°C for 5 s, and a final melting curve. Samples were measured in duplicates using 10 µL of reaction mixture composed of 2.7 µL PCR-grade water, 5 µL KAPA SYBR® FAST qPCR Master Mix (2×) (KAPA Biosystem, Wilmington, United States), 0.3 µL PNA mix (50 µM), 0.5 µL of each primer (10 µM), and 1 µL template DNA. Gene copy numbers (gcn) that were detected in the negative control samples were subtracted from the respective run. Gene copy numbers were calculated per g of sample. It must be noted that this method does not distinguish between viable and non-viable bacterial cells, which should be taken into account when interpreting the results.

### Amplicon library preparation

An amplicon library targeting the variable regions V4 of the 16S rRNA of bacteria was prepared using barcoded 515f and 806r primers (Caporaso et al. 2011). To block the amplification of mitochondrial and plastid RNA of eukaryotic origin, peptide nucleic acid (PNA) oligomers were applied (Lundberg et al. 2013). For each sample, two technical replicates with a reaction volume of 25 µL each [12.4 µL KAPA Taq ReadyMix (2×) (KAPA Biosystem, Wilmington, United States), 0.62 µL of each primer (10 µM), 0.9 µL PNA mix (50 µM) (PNA Bio Inc., Thousand Oaks, United States), 9.46 µL PCR-grade water, and 1 µL template DNA] were prepared. The PCR amplifications were performed with the Eppendorf Mastercycler nexus X2 (Hamburg, Germany) using the following cycling conditions: initial denaturation at 96°C for 3 min, followed by 30 cycles of denaturation at 96°C for 30 s, PNA annealing at 78°C for 5 s, primer annealing at 54°C for 30 s, extension at 72°C for 20 s, and a final elongation for 30 s at 72°C. The technical replicates were combined and the DNA concentration of each sample was determined using the Qubit 4 Fluorometer (Thermo Fisher Scientific, Waltham, United States) and Image Lab Software (Bio-Rad Laboratories, Inc., Hercules, United States) before pooling all samples in equimolar concentration. The amplicon library was purified using the Wizard SV Gel and PCR Clean-Up System (Promega, Madison, WI, United States) and sent for Illumina MiSeq PE 250 sequencing (Novogene, Cambridge, UK).

### Bioinformatic data processing and statistical analysis

Cutadapt was used for demultiplexing of raw sequence data and the removal of primer and barcode sequences (Martin 2011). The discard-untrimmed flag ensured that only reads containing both primer sequences were retained, and no-indels disabled indel matching to increase stringency. DADA2 (Callahan et al. 2016), implemented in QIIME2 (Bolyen et al. 2019), was employed for quality control and the removal of chimeric sequences. DADA2 was run with truncation lengths of 160 base pairs for both forward and reverse reads to remove low-quality regions. Chimera removal was performed using the consensus method integrated into DADA2. The denoising step yielded amplicon sequence variants (ASVs), a feature table, representative sequences, and denoising statistics. Taxonomic assignment of the generated ASVs was performed using the SILVA v138 database (Quast et al. 2013). A total of 18 462 075 bacterial high-quality reads were obtained after quality filtering and removal of non-target (chloroplast and mitochondrial) sequences and assigned to 23 337 ASVs.

Statistical analysis was performed using R (version 4.4.1) (R Core Team 2022) in RStudio version 2023.12.1.402 (Allaire 2012). For bacterial community analysis, the R package phyloseq was used (McMurdie and Holmes 2013). The dataset was rarefied to the lowest read count (28 388 reads) by randomly selecting subsets of sequences. It should be noted that rarefaction discards a portion of sequencing data and thereby may result in the loss of potentially relevant biological information. However, for alpha and beta diversity estimations, which were the scope of the present study, rarefaction remains an accepted approach for standardizing sampling effort (Weiss et al. 2017, Schloss 2024). The rarefied dataset was used for alpha (bacterial richness and diversity) and beta (variation in bacterial composition) diversity analysis. Differences in species richness, Shannon diversity, and evenness between produce, areas and vendors were analyzed using the Kruskal–Wallis test, followed by Dunn’s post-hoc test for multiple comparisons. To analyze differences within the bacterial community structures between produce, areas and vendors, non-metric Bray–Curtis dissimilarity matrices were calculated, and principal coordinate analysis (PCoA) plots were generated for two-dimensional visualization. Statistical differences were assessed using permutational analysis of variance (PERMANOVA, 999 permutations). To assess significant differences in bacterial gene copy numbers between produce, areas and vendors, Kruskal–Wallis tests were employed, followed by multiple pairwise comparisons using Dunn’s post-hoc tests from the R package dunn.test (Dinno 2017). *P*-values were adjusted using Benjamini–Hochberg adjustment.

Cytoscape 3.8.2 software (Shannon et al. 2003 ) was employed for constructing the network of shared and unique ASVs of traditional dried food microbiota, considering only ASVs present in 90% of all sample replicates and with at least 0.01% abundance. The correlation between bacterial richness and total abundance to specific health-relevant taxa was calculated using the Spearman correlation coefficient. To minimize spurious correlation, only ASVs with at least 0.1% relative abundance in the dataset were considered. ASVs classified as *Lactobacillus, Prevotella*, and *Bifidobacterium* were considered as potentially beneficial taxa, and *Salmonella, Vibrio, Klebsiella, Stenotrophomonas*, and *Acinetobacter* as potential opportunistic or environmental human pathogens. This classification should be interpreted with caution, as 16S rRNA gene sequencing does not provide direct information on functional traits or pathogenicity. Rather, this categorization is based on existing literature in which these genera are associated with beneficial (Derrien et al. 2022, Ross et al. 2024, Ofordile et al. 2025) or pathogenic (Bintsis 2017, Li et al. 2019, Hartantyo et al. 2020, Carvalheira et al. 2021) characteristics, respectively.

### Functionality assays of bacteria isolated from traditional dried foods

#### Isolation and identification of bacteria

For the isolation of bacteria from the dried leaf and fruit products, samples were first crushed with mortar, and pestle, dissolved in sterile 0.85% NaCl, and agitated overnight at room temperature. The baobab powder was directly dissolved in sterile 0.85% NaCl and agitated overnight. Serial dilutions of the suspensions were prepared and plated in duplicates on Nutrient Broth II agar (NA) media (SIFIN, Berlin, Germany) and Reasoner’s 2A (R2A) (Carl Roth GmbH + Co. KG; Karlsruhe, Germany). The plates were incubated at room temperature for 48 h. Single colonies were selected based on varying morphology and color and sub-cultured onto NA and R2A plates. For long-term storage at −70°C, overnight cultures of the isolates were prepared in NBII media (SIFIN, Berlin, Germany) and mixed with 50% glycerol in equal volumes.

For taxonomic identification of isolates, genomic DNA was extracted by suspending single colonies in 500 µL rapid lysis buffer (10 mM Tris-HCl, 1 mM EDTA, 1% Triton ×100, ddH_2_O). The suspension was incubated at 95°C for 10 min (Kolia-Diafouka et al. 2018), and genomic DNA was used for subsequent amplification of the 16S rRNA gene using the primer pair 27F and 1492R (Marchesi et al. 1998). PCR products were sent for Sanger sequencing to the commercial sequencing provider LGC Genomics (Berlin, Germany). The quality of the obtained sequences was manually checked using BioEdit (Hall 1999). Quality-filtered sequences were compared against the SILVA database (Quast et al. 2013). A multiple sequence alignment of the sequences was performed, and a phylogenetic tree using maximum likelihood was constructed in MEGA11 (Molecular Evolutionary Genetics Analysis) (Kumar et al. 2018). Visualization of the phylogenetic tree was performed in iTOL (Interactive Tree Of Life) (Letunic and Bork 2021).

#### Antagonistic activity against opportunistic human pathogens

Bacterial isolates were tested for their antagonistic activity against opportunistic human pathogens from the culture collection of the Department of Internal Medicine, Medical University of Graz: *Acinetobacter baumannii* strain 6 340 276, *Enterococcus faecium* strain 6 428 631, *Escherichia coli* strain 6 402 087, *Pseudomonas aeruginosa* strain 6 436 029, and *Stenotrophomonas maltophilia* strain EA23, and against Methicillin-resistant *Staphylococcus aureus* (MRSA) from the culture collection of the Institute of Environmental Biotechnology, Graz University of Technology. NA plates were inoculated with individual opportunistic pathogens, and 5 µL of overnight bacterial cultures were thereafter spotted on the plates. Plates containing only the individual pathogens and only the dried food isolates served as positive and negative controls, respectively. Isolates that produced visible inhibition zones after incubation for 4 days at 25°C were classified as antagonistic against the opportunistic human pathogens (Wicaksono et al. 2022).

#### Biosurfactant-production

Isolates were screened for the production of biosurfactants, which reduce pathogen adhesion to surfaces, using a qualitative drop-collapse method (Bodour and Miller-Maier 1998). For the assay, 2 µL of mineral oil was added into the circular wells of a 96-well plate lid. After equilibration of the mineral oil for 2 h, 5 µL of overnight bacterial culture was added to the wells while holding the pipette at an angle of 45⁰. Bacterial isolates were either classified as positive for the production of biosurfactant if the mineral oil drop collapsed after 1 min or classified as non-biosurfactant-producing if the drop remained beaded.

#### Protease activity

Isolates were screened for protease activity by spotting 5 µL of overnight bacterial culture on 10% skim milk (Heirler Bio Magermilchpulver, Heirler Cenovis GmbH, Germany) agar plates (Pailin et al. 2001). Protease activity was assessed after incubation for 4 days at 25°C. Isolates tested positive for protease activity if a clear hydrolysis zone formed around the isolate. This test has been applied to screen for microbes that disrupt the biofilm development of opportunistic pathogens (Banat et al. 2014, Primo et al. 2015).

#### Bile salt tolerance

Bile salt tolerance was tested to identify strains that survive the gastric passage and might contribute to the diversity of the gut microbiota. Tolerance was determined using a modified version of the spectrophotometric assay described by Prete et al. (2020). In 96-well plates, 5 µL of overnight bacterial culture was added to 200 µL NBII medium with increasing bile salts concentrations (0%, 0.30%, 1.8%, and 3.6% w/v; Thermo Fisher Scientific, Waltham, United States). Following incubation for 24 h at 25°C, bacterial growth was determined by measuring the optical density (OD_600_) using the Tecan microplate reader Infinite® 200 PRO (Tecan Austria GmbH, Grödig, Austria).

#### Antimicrobial resistance

The bacterial isolates were screened against 10 different antibiotics (Ampicillin, Vancomycin, Erythromycin, Tetracycline, Ciprofloxacin, Gentamicin, Kanamycin, Rifampicin, Nalidixic acid, Sulfadiazine). Disk diffusion assays using a fixed antibiotic concentration (20 µg/mL) were applied as a screening approach to identify isolates capable of growth under relatively high antibiotic exposure. Similar screening strategies using elevated antibiotic concentrations have previously been applied in environmental resistome studies to detect strong resistance phenotypes (D’Costa et al. 2006, Walsh and Duffy 2013, Wicaksono et al. 2023b). Briefly, triplicate Mueller–Hinton agar (MHA) plates containing a specific antibiotic at a concentration of 20 µg/mL were inoculated with 3 µL of overnight bacterial culture using a multipoint inoculator and then incubated at 25°C. Control plates without antibiotic supplementation were also included. The inoculated plates were monitored daily for three days. Bacteria demonstrating visible growth on agar plates containing antibiotics from day four of incubation were categorized as resistant. The purpose of this assay was to provide a comparative overview of antibiotic tolerance within the environmental isolate collection rather than to classify isolates according to clinical susceptibility standards.

## Results

### The dried plant product determines bacterial diversity and abundance

**Figure 2:**
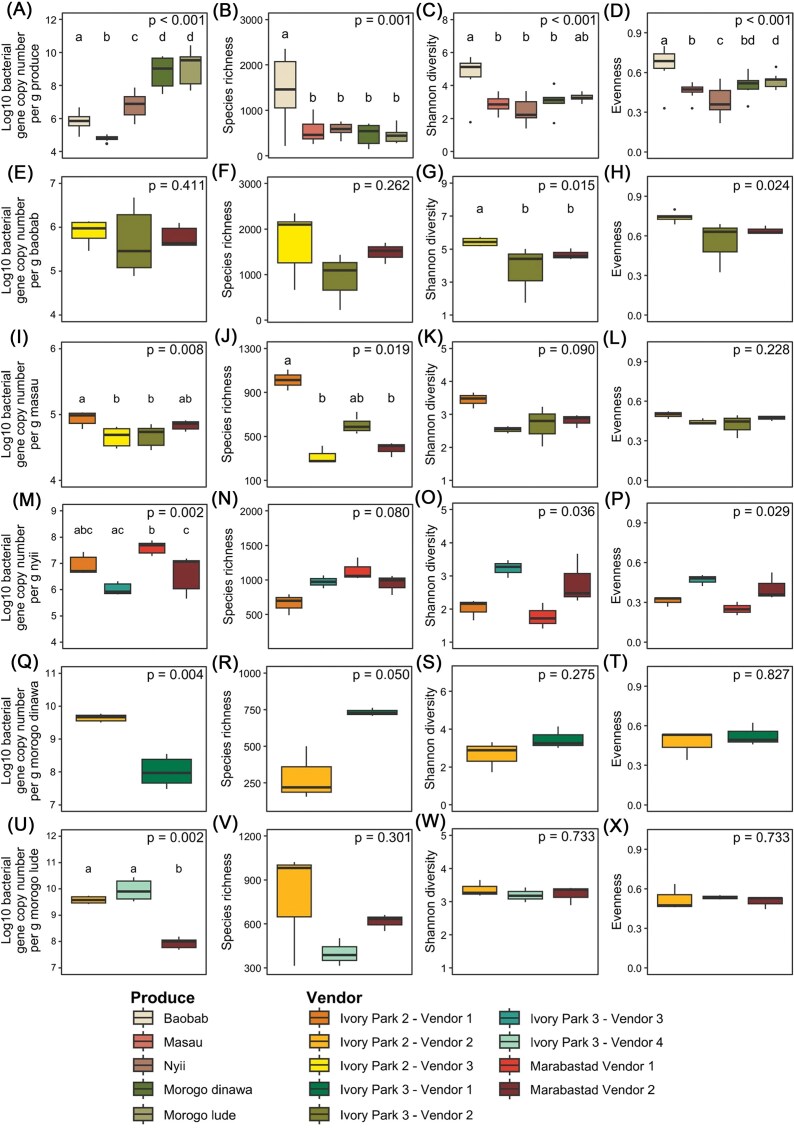
Bacterial abundance and diversity metrics from traditional dried plant products from urban informal markets. Log_10_ bacterial gene copy numbers per gram product were measured via qPCR. Panels (A–D) compare bacterial abundance, species richness, Shannon diversity, and evenness among the traditional dried products, respectively. Panels (E–H) show the same indices for vendor-related impacts only for baobab, panels (I–L) for masau, panels (M–P) for nyii, panels (Q–T) for morogo dinawa, and panels (U–X) show vendor-related impacts on bacterial abundance and diversity for morogo lude. Statistical significance was determined using Kruskal–Wallis test followed by Dunn’s post-hoc tests for pairwise comparisons.

Dried plant products differed significantly in bacterial abundance and diversity. For abundance, a clear pattern emerged concerning the plant materials used for these products. Specifically, the fruits baobab, masau, and nyii showed relatively low bacterial abundance (mean of 5.95 × 10^6^ gcn) compared to leafy greens dinawa and lude (mean of 3.99 × 10^9^ gcn). Regarding diversity estimates, baobab demonstrated significantly higher richness (1458 ASVs), diversity (4.6), and evenness (0.67) compared to all other samples (mean richness: 527 ASVs, mean Shannon diversity: 2.4, mean evenness: 0.5) (Fig. 2A–D). While we found no overall influence of the market, bacterial abundance and diversity of some dried products were affected by the vendor. For baobab, Shannon diversity differed significantly (*P* = 0.015), with “Vendor 3-Ivory Park 2” showing higher diversity than “Vendors 2-Ivory Park 3” and “Vendor 2-Marabastad”. In masau, bacterial abundance was significantly different (*P* = 0.008), being higher in “Vendor 1-Ivory Park 2” compared to “Vendor 3-Ivory Park 2” and “Vendors 2-Ivory Park 3”. Species richness reached the highest value in “Vendor 1-Ivory Park 2” as well, with significant difference to “Vendor 3-Ivory Park 2” and “Vendor 2-Marabastad”. For nyii, bacterial abundance in “Vendor 1-Marabastad” was significantly higher compared to “Vendor 3-Ivory Park 3” and “Vendor 2-Marabastad”. Shannon diversity (*P* = 0.036) and Evenness also differed significantly (*P* = 0.029) among vendors, while no significant differences between individual vendors were identified after correction for multiple testing. In morogo dinawa, “Vendor 3-Ivory Park 3” showed significantly higher abundance than “Vendor 1-Ivory Park 3”, while the opposite pattern was observed for species richness. However, dinawa was only obtained from two different vendors, limiting the robustness of vendor-related statistical findings. For morogo lude, “Vendor 2-Marabastad” showed significantly less bacterial abundance compared to “Vendor 2-Ivory Park 2” and “Vendor 4-Ivory Park 3”, while no significant vendor-specific differences were observed for alpha diversity metrics.

Overall, vendor-specific effects were product-dependent, and no consistent pattern was observed indicating that a particular vendor was systematically associated with higher or lower diversity metrics across products. These product-specific differences in bacterial abundance and diversity were further investigated by analyzing differences in bacterial community composition among dried plant products.

### Dried plant product determines bacterial composition

**Figure 3:**
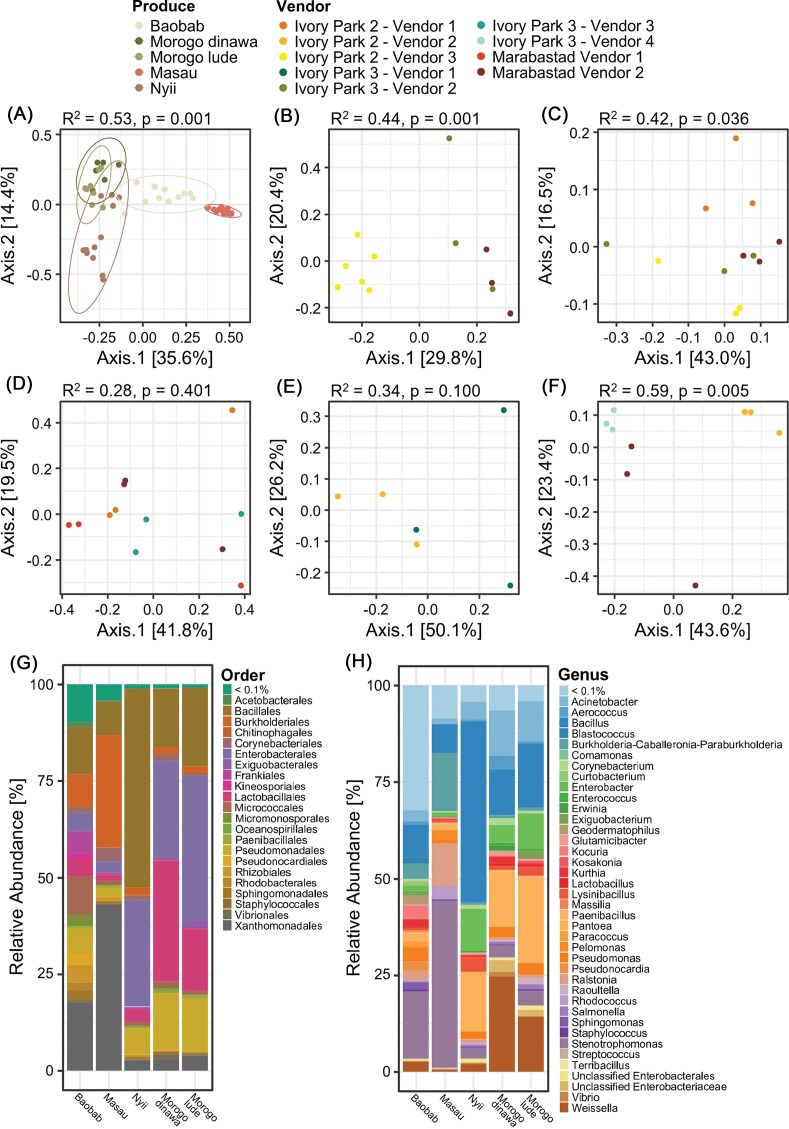
Bacterial community structure and composition of traditional dried plant products obtained from urban informal markets. PCoA plots (A–F) are based on Bray–Curtis dissimilarity matrix and statistical significance was assessed by PERMANOVA analysis. Samples grouped by dried produce type are shown in (A). The samples of the individual produce types baobab (B), masau (C), nyii (D), morogo dinawa (E), and morogo lude (F) are grouped by the vendors. The bar charts depict relative abundance of bacteria on genus level (G) and order level (H) in each dried product.

Dried plant products differed significantly in the bacterial community composition, explaining over 53% of the differences (*P* = 0.001). In conformity with the measurements for bacterial abundance and diversity, the plant material played a role, with the two types of leafy greens (dinawa and lude) clustering close together in the PCoA plot (Fig. 3A). Vendors had a product-specific impact for baobab, 44% of the differences in the bacterial composition were determined by the vendor (Fig. 3B). For masau (Fig. 3C) and morogo lude (Fig. 3F), the vendor determined 42% and 59%, respectively. No significant impact of the vendor was observed for dinawa and nyii (Fig. 3D and E), however, only two different vendors offered dinawa, thus, the statistical robustness of this calculation is limited.

The bacterial communities of all dried products were dominated by Proteobacteria (55%), followed by Firmicutes (35%), and Actinobacteria (9%). Differences in bacterial taxonomy were apparent at the order level, where baobab was dominated by Xanthomonadales (18%), Bacillales (12%), and Micrococcales (10%), masau was dominated by Xanthomonadales (43%), Burkholderiales (29%), and Bacillales (9%), nyii was dominated by Bacillales (52%), Enterobacteriales (28%), and Pseudomonadales (4%), dinawa was dominated by Lactobacillales (31%), Enterobacteriales (25%), and Bacillales (15%), and lude was dominated by Enterobacteriales (37%), Bacillales (20%), and Lactobacillales (16%) (Fig. 3G). At the bacterial genus level, baobab was dominated by a high number of rare taxa (<0.1% relative abundance), followed by *Stenotrophomonas* (17.3%) and *Bacillus* (8.5%). The most abundant genera in masau were *Stenotrophomonas* (42.8%), *Burkholderia-Caballeronia-Parabulkholderia* (15%), and *Ralstonia* (11.1%). *Bacillus* (47%), *Pantoea* (15%), and *Enterobacter* (10.9%) were most abundant in nyii. The leafy greens dinawa and lude were dominated by *Weisselia* (24.7%) and *Pantoea* (21.7%), respectively (Fig. 3H).

Given the observed differences in bacterial composition among products, a core microbiome analysis was conducted to identify taxa shared across products as well as those unique to specific plant types.

### Core microbiome composition of traditional dried plant foods

**Figure 4:**
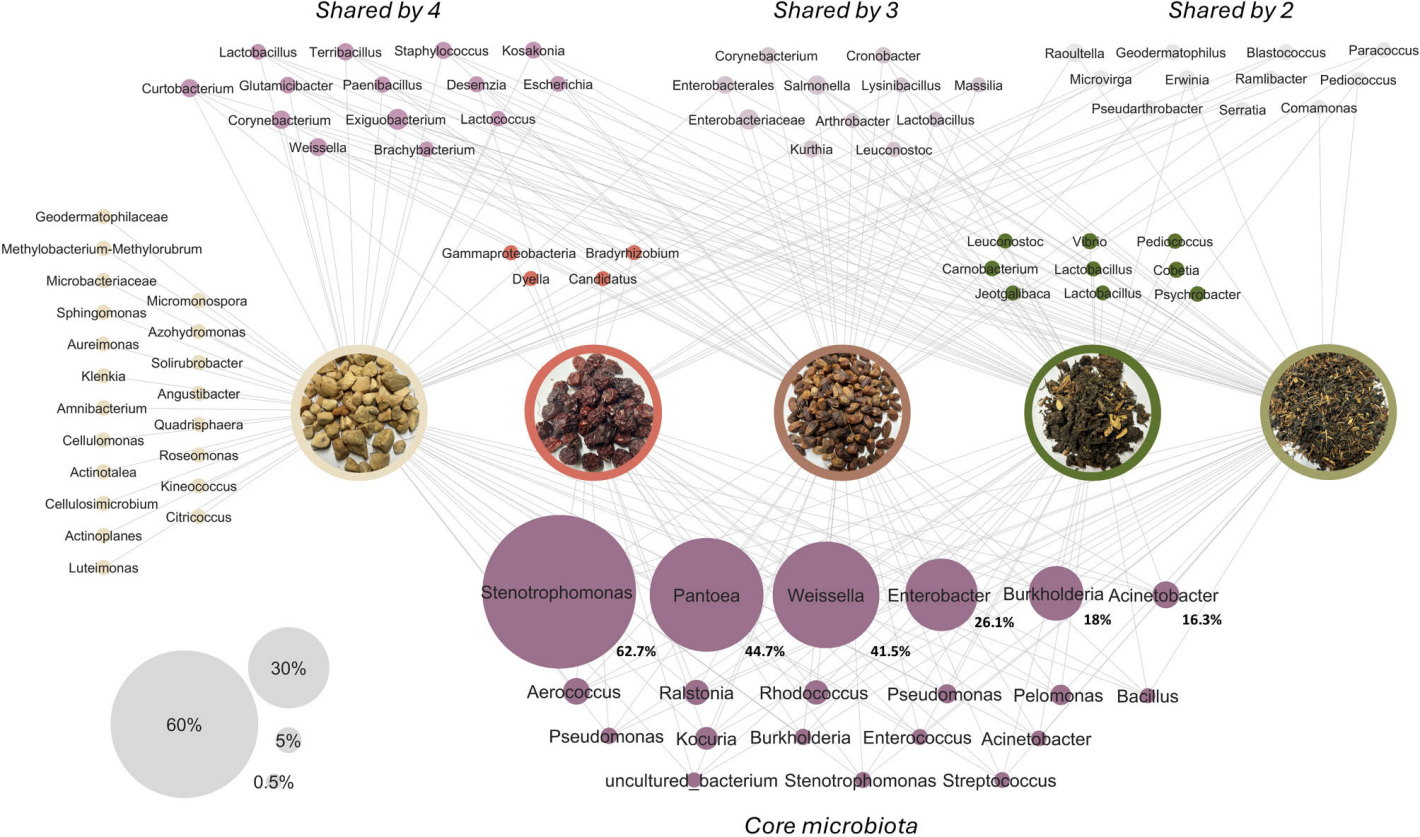
Core bacteria of traditional dried plant products baobab, masau, nyii, lude, and dinawa (from left to right) highlighting common and unique ASVs. Node size corresponds to relative abundance (%) of ASVs in the dataset, as indicated in the legend on the lower left, and percentage values of highest abundant core microbiota are added in addition. Only ASVs present in 90% of all dried product sample replicates and with at least 0.01% abundance were included. The ASVs assigned to Burkholderia-Caballeronia-Paraburkholderia were abbreviated as *Burkholderia* for simplicity.

A core microbiome analysis was conducted to identify shared and unique ASVs in traditional dried foods (Fig. 4). The core microbiome consisted of 20 ASVs, of which *Stenotrophomonas* (62.7%), *Pantoea* (44.7%), *Weissella* (41.5%), *Enterobacter* (26.1%), *Burkholderia* (18%), and *Acinetobacter* (16.3%) were most abundant. All of these ASVs, with the exception of *Weissella*, represent genera that include diverse environmental bacteria but may also contain opportunistic pathogenic species. However, because taxonomic assignments were based on 16S rRNA gene sequencing, it is not possible to determine the presence of pathogenic strains or virulence traits. Baobab contained the highest number of unique ASVs (20 ASVs), followed by lude (9 ASVs), and masau (4 ASVs). No unique ASVs were found in nyii and dinawa. These unique ASVs, as well as those that were shared by four (14 ASVs), three (11 ASVs), and two (11 ASVs) dried products were mostly low abundant, exhibiting between 0.01% and 7.6% abundance. These findings highlight both shared and product-specific bacterial taxa across dried plant products. To further examine relationships between selected taxa and overall microbial patterns, correlations between bacterial richness, total abundance, and selected genera were assessed.

### Associations between bacterial richness, total abundance, and selected taxa

Spearman correlation analysis was performed on ASVs assigned to taxa reported in the literature as either potentially beneficial [*Lactobacillus, Bifidobacterium, Prevotella* (Derrien et al. 2022, Ross et al. 2024, Ofordile et al. 2025)] or associated with foodborne or opportunistic pathogenic taxa [*Salmonella, Vibrio, Klebsiella, Acinetobacter, Stenotrophomonas* (Bintsis 2017, Li et al. 2019, Hartantyo et al. 2020, Carvalheira et al. 2021)]. Only taxa that reached at least 0.1% relative abundance in the dataset were included in the analysis. *Bifidobacterium* and *Prevotella* showed positive correlations with bacterial richness, whereas *Acinetobacter* showed a negative correlation with richness. In contrast, when considering absolute bacterial abundance determined by qPCR, *Bifidobacterium* and *Prevotella* were negatively correlated with higher bacterial loads, while *Lactobacillus, Salmonella, Vibrio*, and *Acinetobacter* showed positive correlations with increasing bacterial abundance (Table 1). Importantly, there correlations are based solely on taxonomic profiles derived from 16S rRNA gene sequencing and do not allow conclusions regarding functional traits or health-related effects.

**Table 1:** Spearman correlation coefficient analyses of potentially health-relevant bacteria to richness and total abundance (16S rRNA gcn).

	Richness (No. ASVs)		Abundance (16S rRNA gcn)	
Taxa of potential health relevance	*r*	*P*	*r*	*P*
*Lactobacillus*	−0.22	0.11	**0.46**	**<0.001**
*Bifidobacterium*	**0.56**	**<0.001**	**−0.45**	**<0.001**
*Prevotella*	**0.39**	**0.005**	**-0.39**	**0.005**
*Salmonella*	−0.16	0.27	**0.64**	**<0.001**
*Vibrio*	0.038	0.79	**0.4**	**0.004**
*Klebsiella*	0.16	0.27	−0.005	0.97
*Acinetobacter*	**−0.28**	**0.05**	**0.71**	**<0.001**
*Stenotrophomonas*	−0.03	0.83	−0.23	0.11

Significant correlations are highlighted in bold.

To complement sequencing-based observations, cultivation-dependent assays were conducted to evaluate selected functional traits of representative bacterial isolates.

### Functional traits of bacterial isolates from traditional dried plant products

**Figure 5:**
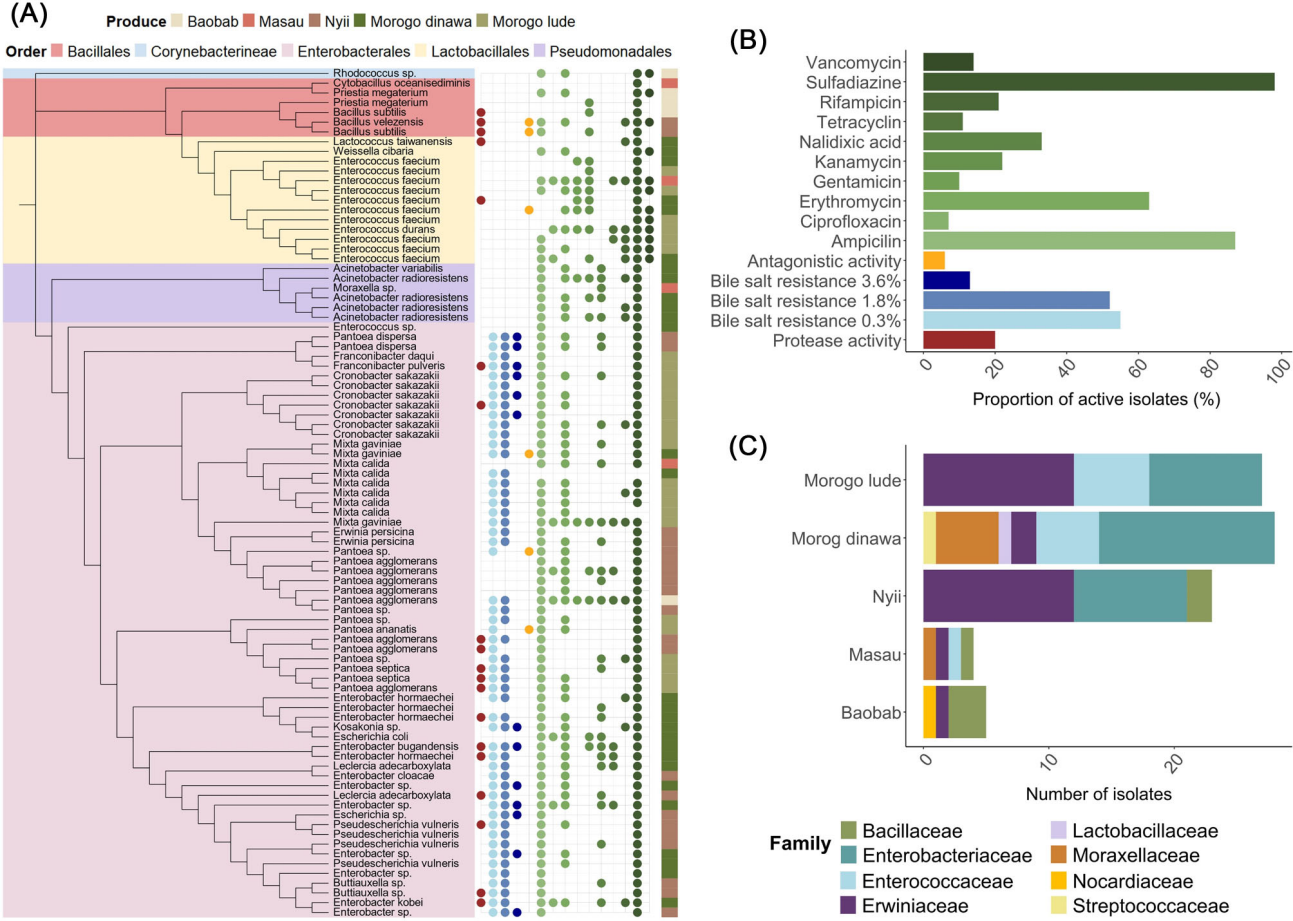
Functional characteristics of dried product-associated bacterial isolates. Phylogenetic tree (A) is based on partial 16S rRNA genes of bacterial isolates. Each isolate is annotated with its genus/species name, and colored squares on the right indicate the source produce type (top legend). The tree is color-coded by bacterial order (top legend). Functional traits and antibiotic resistance phenotypes of each isolate are shown as colored dots in the matrix. These include protease activity (red dots), bile salt resistance at three concentrations (light to dark blue dots), antagonistic activity against selected opportunistic human pathogens (yellow dots), and phenotypic resistance to nine antibiotics (green dots). The color code for the functional traits corresponds to panel (B), which shows the proportion (%) of bacterial isolates exhibiting activity in each functional assay. Panel (C) displays the distribution of isolates by produce type, with bars indicating the number of isolates per product and colors representing different bacterial families.

A total of 87 bacteria were isolated from the traditional dried foods (baobab *n*= 5, masau n= 4, nyii *n*= 23, dinawa *n*= 28, and lude *n*= 27), belonging to the bacterial orders Enterobacterales (*n*=60), Lactobacillales (*n*=14), Bacillales (*n*=6), Pseudomonadales (*n*=6), and Corynebacterineae (*n*=1) (Fig. 5C). Isolates were taxonomically identified by comparing sequences derived from Sanger sequencing against the SILVA database. It must be noted that the 16S gene is often not sufficient to identify the species, especially when species are closely related, thus species identity is putative and based on the best match in the reference database. Cultivation-dependent assays were conducted to screen bacterial isolates for functions potentially relevant to human health. These functions included protease activity, antagonistic activity against selected human pathogens, tolerance to bile salts, biosurfactant production, and phenotypic resistance to 10 different antibiotics representing seven drug classes (Fig. 5).

Protease activity was confirmed for 19 isolates. Enterobacteriales isolates showed tolerance to bile salts at concentrations of 0.3% (53 isolates), 1.8% (50 isolates), and 3.6% (13 isolates). Antagonistic activity against methicillin-resistant MRSA was observed for the isolates *Bacillus velezensis* (nyii), *Bacillus subtilis* (nyii), *Enterococcus faecium* (dinawa), *Pantoea ananatis* (lude), and *Mixta gaviniae* (dinawa). Growth of *E. coli* 6 402 087 was inhibited by *Pantoea ananatis* (lude) and *Pantoea sp*. (nyii), and *S. maltophilia* EA23 was inhibited by the isolate *Bacillus subtilis* (nyii). None of the isolates showed antagonistic activity against *A. baumannii* 6 340 276, *E. faecium* 6 428 631, and *P. aeruginosa* 6436029. No biosurfactant production was detected among the tested isolates (Fig. 5A).

In phenotypic antimicrobial susceptibility assays, a high proportion of isolates exhibited growth in the presence of sulfadiazine (*n* = 85) and ampicillin (*n* = 84), followed by erythromycin (*n* = 55). Growth in the presence of other antibiotics included kanamycin (*n* = 19), nalidixic acid (*n* = 29), and rifampicin (*n* = 18), while fewer isolates tolerated ciprofloxacin (*n* = 6), gentamicin (*n* = 9), tetracycline (*n* = 10), and vancomycin (*n* = 12) (Fig. 5A and B). These results indicate phenotypic tolerance under the tested conditions, which included a fixed antibiotic concentration (20 µg/mL) and represent a method applied in environmental resistome studies to detect strong resistance phenotypes (D’Costa et al. 2006, Walsh and Duffy 2013). The presence of specific antimicrobial resistance genes or clinically relevant resistance mechanisms was not assessed in this study.

Overall, cultivation-based assays demonstrated that selected isolates exhibited diverse phenotypic traits under laboratory conditions providing complementary insights to sequencing-based observations of microbial diversity associated with traditional dried plant foods. It should be noted that these assays were performed on a limited number of cultured isolates relative to the total number of ASVs detected in the sequencing dataset and therefore do not represent the full functional potential of the microbial communities associated with these foods.

## Discussion

Our analysis of traditional dried plant products collected from urban informal markets in Pretoria and Johannesburg showed that bacterial diversity was generally consistent across samples, except baobab, which exhibited significantly higher diversity. Bacterial communities clustered according to plant type, with notable similarities among fruit-derived and leafy green products. Vendors accounted for up to 59% of the observed variation in bacterial community composition between products. However, vendor-specific effects were product-dependent, and no consistent pattern was observed indicating that a particular vendor was systematically associated with higher or lower bacterial abundance or diversity across products. Furthermore, the number of vendors available for certain products was limited, which reduced the statistical power to detect vendor-related effects. Phenotypic assays of cultured isolates revealed widespread growth in the presence of multiple antibiotics, particularly among isolates obtained from dried leafy greens. Phenotypic screening was, however, conducted on a limited number of isolates relative to the total bacterial richness detected in the sequenced dataset. Our findings suggest that drying techniques, postharvest handling, and environmental exposure may represent important ecological drivers shaping microbial community structure, although the specific contributing factors could not be directly resolved within the scope of this study.

Drying is among the most widely employed food preservation strategies, significantly reducing microbial loads and thereby extending shelf life (Santos et al. 2025). In the present study, bacterial loads ranged from 10³ gcn g^−1^ in masau to 10⁹ gcn g^−1^ in dinawa and lude, which is comparable to those in fresh tissues (e.g. apple fruit pulp showing ∼10⁵ gcn g^−1^ (Wassermann et al. 2019)). However, the methodology used here does not distinguish between viable and non-viable bacterial cells, and therefore the number of living bacteria may be lower than the values reported. Previous studies applying viability-PCR approaches have demonstrated that a portion of bacterial DNA detected in dried foods originates from dead cells (Wicaksono et al. 2022). Specific drying conditions are also likely to influence microbial survival. Traditional sun- or air-drying methods commonly used by vendors at informal markets may involve lower temperatures and less controlled environmental conditions than industrial dehydration, potentially allowing a larger fraction of microorganisms to persist (Dhaliwal et al. 2021, Prestes et al. 2025). The absence of standardized drying protocols therefore likely represents an important factor influencing microbial loads and community composition. In addition to the drying technique, handling practices such as washing procedures, drying surfaces, storage containers, exposure to dust or insects, and personal hygiene during product handling could all affect microbial transfer and survival. However, these variables were not systematically recorded and should be addressed in future investigations. Another methodological aspect relates to sample preparation prior to DNA extraction. For the dried fruits nyii and masau, samples were soaked overnight in sterile sodium chloride solution to facilitate seed removal and ensure that only microbiota associated with the edible pulp were analyzed. Although soaking can influence microbial composition (Kim et al. 2022), the bacterial communities of masau and nyii remained clearly distinct in both composition and abundance, suggesting that the microbiota of the raw plant material plays a major role in shaping the final dried product.

Among all dried products, baobab, which is widely recognized for its nutritional value (Foltz et al. 2021, Duysburgh et al. 2024) and traditionally used to address malnutrition in infants (Asogwa et al. 2021), exhibited the highest bacterial diversity, richness, and evenness, and the second lowest abundance after masau. In contrast, the two dried leafy green products, dinawa and lude, exhibited the lowest bacterial diversity, richness, and evenness, and the highest bacterial abundance. In our dataset, high bacterial richness was positively correlated with the relative abundance of genera such as *Bifidobacterium* and *Prevotella*; genera that include taxa previously reported in beneficial microbial contexts (Derrien et al. 2022, Ofordile et al. 2025). High abundance correlated with the relative abundance of genera such as *Salmonella, Vibrio*, and *Acinetobacter*, which include species reported in foodborne or environmental contexts (Bintsis 2017, Carvalheira et al. 2021, Prestes et al. 2025). Moreover, the core microbiome shared across the dried plant foods contained genera that include clinically relevant species, such as *Stenotrophomonas* and *Acinetobacter*, which are nevertheless commonly detected in environmental microbiomes, including those associated with plants (Ryan et al. 2009, Berg and Martinez 2015). Importantly, interpretations informed by previous literature are intended to provide contextual support rather than to imply direct functional inference from the present dataset, as this study is based solely on genus-level taxonomic data and does not allow functional or health-related conclusions. Confirming such characteristics would require higher-resolution approaches, such as metagenomic sequencing or targeted PCR assays for virulence-associated genes and functional capabilities (Beck et al. 2021, Pham et al. 2025). Moreover, the extent to which microbes from dried plant foods survive processing, gastrointestinal passage, or influence the human gut microbiome remains unresolved. Therefore, any potential interaction between these food-associated microbes and the gut microbiome should be considered hypothetical.

In general, the plant microbiome is plant species-specific, plant tissue-specific, and affected by environmental factors and agricultural management practices (Trivedi et al. 2020). Those microbes play fundamental roles for growth, development, resistance, and evolution of the host plant (Vandenkoornhuyse et al. 2015, Berg et al. 2020). In the present study, the plant species accounted for 53% of the variation between products, which may reflect intrinsic characteristics of the plant matrix, including surface morphology, chemical composition, nutrient availability, and antimicrobial secondary metabolites (Bouwmeester et al. 2025). For example, leafy greens provide large exposed surface areas with stomata and trichomes that can facilitate microbial attachment and colonization, whereas fruit tissues often contain higher concentrations of sugars, acids, and phenolic compounds that can selectively favor certain microbial groups (Vorholt 2012). In the present study, the dried leafy green products were dominated by Enterobacteriales (25% and 37% rel. abundance in dinawa and lude, respectively), which are common inhabitants of the phyllosphere (Vorholt 2012, Leff and Fierer 2013, Cardinale et al. 2015, Cernava et al. 2019). Their presence may therefore reflect natural plant-associated microbiota rather than contamination alone. Nevertheless, leafy vegetables have previously been associated with foodborne outbreaks, often linked to irrigation water quality, harvesting conditions, and postharvest handling practices (Jongman and Korsten 2016, Richter et al. 2021).

Although enterobacteria can be associated with foodborne risks, some studies have reported beneficial functions such as vitamin B production and immunomodulatory effects (Sokolowska et al. 2018, , Vatanen et al. 2016 , Wicaksono et al. 2024). Consistent with this functional diversity, several enterobacteria isolates in this study showed protease or antagonistic activity against tested pathogens. At the same time, Enterobacteriales dominated the isolates that showed growth in the presence of multiple antibiotics, most frequently sulfadiazine and ampicillin, under the tested conditions. Such observations may reflect ecological characteristics of plant-associated microbiomes, which can include both β-lactam-producing microorganisms and microorganisms with mechanisms conferring tolerance to these compounds (Obermeier et al. 2021, Gholipour et al. 2024), as well as selective pressures associated with antimicrobial secondary metabolites produced in plant environments (Berg et al. 2005, Cernava et al. 2019, Chen et al. 2019, Brennan et al. 2022). After Enterobacteriales, Lactobacillales isolates presented the second largest spectrum of antimicrobial resistance in the present dataset, which aligns with recent studies on the antimicrobial resistance profiles of Lactobacillales (Kaszab et al. 2023, Luo et al. 2024). Importantly, assays were conducted at a fixed antibiotic concentration (D’Costa et al. 2006) without determination of minimal inhibitory concentration or CLSI breakpoints, thus, the results should be interpreted as phenotypic tolerance under the tested conditions rather than clinically defined resistance. Moreover, phenotypic resistance observed in environmental isolates does not necessarily indicate the presence of transferable antimicrobial resistance genes or imply a direct transmission risk to humans. Confirming such risks would require genomic analyses targeting specific resistance determinants and mobile genetic elements.

Several limitations should be considered when interpreting the results of this study. First, the number of vendors per product was limited, which restricts the generalizability of vendor-related effects. Second, metadata on drying conditions, storage practices, and hygiene procedures were not systematically recorded. Third, the 16S rRNA gene sequencing approach used provides limited taxonomic resolution and does not allow functional inference. Fourth, only a limited number of isolates were analyzed relative to the total microbial diversity detected by sequencing, thus these findings should not be interpreted as representative of the entire microbial community. Finally, the study focused exclusively on bacterial communities, whereas fungi and other eukaryotic microorganisms may also contribute to the microbiology of dried foods and their potential interactions with consumers. A methodological limitation relates to sequencing depth normalization. In this study, rarefaction to the lowest read count was applied prior to alpha and beta diversity analyses. Although rarefaction standardizes sampling depth, it has been criticized for discarding data and potentially reducing statistical power. Alternative normalization strategies, such as variance-stabilizing or compositional approaches, have been proposed, particularly for differential abundance testing. Nevertheless, rarefaction is considered suitable for comparing ecological diversity patterns when differential abundance testing is not the primary objective (Weiss et al. 2017, Schloss 2024).

Traditional indigenous foods are typically cultivated, harvested, and processed in ways that differ substantially from those in the formal commercial food sector (da Silva et al. 2025). To better understand potential health implications of dried plant foods, future studies should include direct comparisons between the wide range of traditional dried foods with their commercially processed counterparts. Larger sampling schemes, detailed metadata on handling and drying conditions, and higher-resolution genomic approaches are needed to better understand the microbial ecology of traditional dried foods. In addition, developing structured drying protocols and providing hygiene guidance for vendors could improve microbial quality while preserving the cultural and nutritional value of these foods. Education initiatives focusing on microbiome literacy and food safety may further support safe handling practices within informal food systems (Timmis et al. 2019). Science communication initiatives, e.g. the science pedagogy video game “Tiny Biome Tales” (https://microbiome.gamelabgraz.at/) (Schweitzer et al. 2024), can be leveraged to raise awareness about food safety and at the same time highlight the nutritional and cultural value of traditional plant products and preparation methods.

## Conclusion

The findings of this study indicate that microbial diversity and composition in traditional dried plant products from urban informal markets in South Africa are shaped by multiple interacting ecological drivers, including plant characteristics, environmental exposure during drying, and vendor-specific handling practices. From a food safety perspective, variability introduced during informal processing and storage may represent an important source of microbial heterogeneity, highlighting the relevance of improved hygiene, drying standardization, and controlled storage conditions.

## Supplementary Material

xtag026_Supplemental_File

## Data Availability

The amplicon sequences are available in the European Nucleotide Archive (www.ebi.ac.uk/ena) under the BioProject accession number PRJEB96289.
