# Supplementary material for: Diversity and selected functional traits of microbiota associated with traditional dried plant foods from South African informal markets
Source: FEMS Microbes. 2026 May 13;7:xtag026. doi: 10.1093/femsmc/xtag026 (PMC13220767; doi:10.1093/femsmc/xtag026)
Supplement: xtag026_Supplemental_File [file xtag026_supplemental_file.docx]

**Reviews of manuscript FEMSMC-2026-008.R2**

**Reviewer 1: Nazir Ahmad, Nucl Inst Agr NIA**

**Recommendation: Reject**

• Repeatedly refer to Acinetobacter baumannii, Stenotrophomonas maltophilia, Salmonella, Vibrio however 16S V4 region cannot reliably resolve species SILVA assignments at species level are probabilistic and acknowledged but still interpret them as confirmed pathogens

• Claiming core microbiome members as specific pathogens (e.g., A. Baumannii) may be considered overstated without Whole genome sequencing, Species-specific PCR, MLST and functional virulence assays. This triggers strong reviewer criticism.

• Rarefaction was lowest read count so better alternatives, deseq2 variance stabilization

• Compositional methods (CLR transformation), ANCOM

• Disk diffusion with 20 µg/ml fixed concentration, No CLSI breakpoints, No MIC determination limit clinical relevance.

• Minor but frequent typographical inconsistencies, taxonomic formatting issues, some long sentences need tightening

Need to avoid definitive species claims. Focus on vendor hygiene impact, handling practices and need for structured drying protocols

**Reviewer 2**

**Recommendation: Minor Revision**

General comments

The manuscript “Microbial diversity and health implications of traditional dried plant foods from South African informal markets” aims to assess the potential health implications associated to the microbiota of five traditional dried plant products (baobab, masau, nyii, dinawa and lude) sourced from three informal markets in South Africa, using 16S rRNA gene sequencing, quantitative real-time PCR, and functional assays. Although the study presents results that may be of interest to the field, some points require clarification and review before the manuscript can be considered for publication.

#1. The results indicate that plant type is a determinant of community structure; however, this finding is presented mainly in a descriptive manner. I suggest expanding the discussion to include possible underlying mechanisms, such as differences in plant surface structure, chemical composition of the plant matrix, and other selective factors.

#2. The limitations section is present but could be more comprehensive. The authors are encouraged to further discuss methodological and analytical constraints.

#3. In several instances, statistical significance is reported without clearly indicating which group presented higher or lower values. The direction of the observed effects should be explicitly stated in the text.

#4. Please, ensure a consistent format for figure citations throughout the manuscript (e.g., Figure 3A instead of Figure 3, A).

#5. Statements regarding potential health benefits should be presented more cautiously and framed as hypotheses rather than implications supported by the current data.

#6. A general revision of the English language is required, as the manuscript contains typographical and grammatical errors.

Specific comments

#1. L118: “…interchangeably when referring to dinawa and lude” → “…interchangeably when referring to dinawa and lude, respectively”

#2. L199: “Thermo Fischer Scientific” → “Thermo Fisher Scientific”

#3. L239: Include reference to the SILVA classification tool. The same applies to L259.

#4. L314: At its first occurrence in the text, the acronym GCN should be written in full.

#5. L315: 3.99 x 109?? Please specify the unit.

#6. L390-401: Please cite the figure where these results can be observed.

#7. L426: Wicaksono et al. (Wicaksono et al., 2022) → Wicaksono et al. (2022). Please check the entire manuscript to ensure that references follow the journal style.

#8. L542: “and and assessing their potential transmission via plant-based foods” → “and assessing their potential transmission via plant-based foods”

#9. Figure 2: The description of Panels G-H is missing.

#10. Figure 3: I suggest separating panels 3G–H and presenting them as a new figure to improve readability.

#11. English language and typographical errors:

Examples include:

Paneals → Panels

Panals → Panels

eveneness → evenness

barcharts depicts → bar charts depict

Kruskal Wallis test →Kruskal–Wallis test

Please perform a thorough language revision throughout the manuscript.

**Reviewer 3**

**Recommendation: Major Revision**

Title

The title is clear and appropriate for the scope of the journal. However, the expression “health implications” may overstate the strength of the evidence presented, as the study is largely descriptive and based on 16S rRNA gene sequencing and in vitro assays. The wording should be softened to avoid implying direct health effects.

Abstract

The abstract overinterprets associative findings. Correlations between richness and certain genera are framed as health-relevant implications without sufficient functional validation. The identification of Acinetobacter baumannii and Stenotrophomonas maltophilia as core microbiome members should be contextualized more cautiously, given the taxonomic resolution limits of 16S rRNA sequencing. The sample size and total number of samples analyzed should be clearly stated. Statements suggesting probiotic potential should be moderated.

Introduction

The research question is not explicitly formulated and should be more clearly defined. The conceptual framing of “beneficial” versus “pathogenic” taxa is introduced without sufficient explanation, which later affects the interpretation of results. A clearer distinction between ecological description and health inference is needed. Furthermore, this section could contextualize the products that will be evaluated. Some information is included in the materials and methods section, but for the reader, it is better to be here.

Materials and Methods

The decision to rarefy the dataset should be better justified, considering current debates on rarefaction versus alternative normalization approaches. The criteria used to define genera as “beneficial” or “potential human pathogens” are overly simplistic and require stronger justification or rephrasing. The lack of viability assessment significantly limits the interpretation of absolute abundance data, and this limitation should be emphasized earlier and more clearly. The limited number of vendors for certain products weakens statistical power and should be addressed more transparently.

Results

Several interpretations go beyond what the data support. Correlations between richness and “beneficial taxa” are presented in a way that suggests functional consequences, which cannot be inferred from 16S data alone. The concept of a “core microbiome” dominated by potential pathogens requires more careful discussion, especially given species-level uncertainty. The functional assays were conducted on a limited number of isolates relative to total ASVs, and this limitation should be clearly acknowledged when concluding functional potential. The antimicrobial resistance results are important but should avoid implying direct public health risk without genetic confirmation of resistance determinants.

Discussion

The discussion contains speculative elements that extend beyond the presented evidence. The dichotomous framing of taxa as beneficial versus pathogenic oversimplifies microbial ecology and should be revised. Statements implying possible modulation of the human gut microbiome by these foods are hypothetical and should be clearly labeled as such. The interpretation of antimicrobial resistance findings would benefit from a more cautious tone, emphasizing that phenotypic resistance in environmental isolates does not necessarily equate to transmission risk. Some arguments rely on comparisons with fermented foods or industrial systems that are not directly tested in this study and should be reframed as perspectives rather than conclusions.

Conclusion

The conclusion overstates the health-related implications of the findings. It should focus more strictly on the ecological characterization of microbiota and the need for improved hygiene practices, rather than implying potential health benefits or risks that were not directly measured. The wording should reflect the exploratory and descriptive nature of the study.

**Associate Editor Recommendation: Major Revision**

Please decrease the similarity level with other papers in your text.

**EIC Decision: Major Revision**

**Author's Response to Decision Letter for (FEMSMC-2026-008)**

Editor´s comment 1: You will see that the reviewers, although they found merit in your study, have raised a number of concerns that preclude acceptance in its present form. Specifically, pay close attention to comments related to the rigor of the 16S data analysis and statements with perceived overclaiming interpretation. If you feel able to address these comments and the points mentioned below, then we will be happy to consider a revised manuscript.

Response: Dear Editor, dear reviewers,

We sincerely thank you for the careful evaluation of our manuscript. We appreciate the constructive and detailed comments provided, which have helped us to substantially improve the rigor, clarity, and interpretation of our study. We have carefully addressed all reviewer and editorial comments and revised the manuscript accordingly.

In particular, we paid close attention to concerns regarding the rigor of the 16S rRNA gene data analysis and potential overinterpretation of taxonomic findings. We have comprehensively revised the manuscript to remove statements that could be interpreted as overclaiming. We also revised the title of the manuscript and the featured image, which now focuses on the ecological importance, the impact of the vendor and food safety, instead for overclaiming health effects. All species-level assignments derived from the amplicon dataset were removed, and interpretations are now restricted to the genus level. Statements implying the presence of specific pathogenic species were replaced with more cautious wording. In the Methods section, we clarified that 16S rRNA gene sequencing does not provide information on functional traits or pathogenicity, and that the categorization of taxa as potentially beneficial or pathogenic is based on literature in which these genera are commonly associated with such characteristics. We further specified that such assignments should be considered probabilistic. The Results section was substantially revised and sentences suggesting functional potential were removed. In the Discussion, we explicitly acknowledge the limitations of 16S rRNA gene sequencing and emphasize that conclusions regarding pathogenicity or virulence are not possible.

As all reviewers raised related concerns regarding interpretation of 16S-based results, this clarification is provided in multiple responses to ensure transparency and consistency in addressing this key issue.

Editor´s comment 2: Please decrease the similarity level with other papers in your text.

Response: The manuscript has been carefully revised to reduce textual similarity with previously published work. Several sections, particularly the Introduction and Discussion were rephrased. In addition, redundant phrasing and extended literature descriptions were shortened or rewritten. These revisions improved both the originality and clarity of the manuscript.

Reviewers' Comments to Authors:

Reviewer: 1

Comments to the Author

1.1 Repeatedly refer to Acinetobacter baumannii, Stenotrophomonas maltophilia, Salmonella, Vibrio however 16S V4 region cannot reliably resolve species SILVA assignments at species level are probabilistic and acknowledged but still interpret them as confirmed pathogens

1.1 Response: First, we would like to think the reviewer for the careful assessment of our manuscript and the helpful suggestions provided. In response, the manuscript has been revised to address the points raised and to enhance the clarity, rigor, and transparency of the study. Detailed responses to each comment are provided in the following sections. Regarding the first comment: We fully agree that SILVA assignments at species level are probabilistic and should not be acknowledged as confirmed pathogens. Please see our response to your next comment.

1.2 Claiming core microbiome members as specific pathogens (e.g., A. Baumannii) may be considered overstated without Whole genome sequencing, Species-specific PCR, MLST and functional virulence assays. This triggers strong reviewer criticism.

1.2 Response: We agree with the reviewer and have revised the manuscript to avoid overinterpretation of taxonomic assignments based solely on 16S rRNA gene sequencing. All species level-assignments from the amplicon dataset were deleted from the manuscript, focusing now only on genus level. Statements implying the presence of specific pathogenic species were replaced with more cautious wording. These revisions were implemented in the Abstract, Materials and Methods, Results, Discussion, and Conclusion. In the Methods section, we clarified that 16S rRNA gene sequencing does not provide information on functional traits or pathogenicity, and that the categorization of taxa as potentially beneficial or pathogenic is based on literature where these genera are commonly associated with such characteristics. We added that such assignments are probabilistic and not definitive at the species level (L222 - L229). The Results sections were strongly revised (L341-367), including the header and the corresponding table. All sentences suggesting functional potential were removed. In the Discussion we explicitly acknowledge the limitations of 16S rRNA sequencing and emphasized that conclusions regarding pathogenicity or virulence would require higher-resolution approaches such as whole-genome sequencing or targeted molecular assays (L464-469). We also revised the title of the manuscript as well as the featured image, which now focuses on the ecological importance, the impact of the vendor and food safety, instead for overclaiming health effects.

1.3. Rarefaction was lowest read count so better alternatives, deseq2 variance stabilization Compositional methods (CLR transformation), ANCOM

1.3. Response: We agree about the importance of this point. In this study, rarefaction to the lowest read count (28,388 reads – now also included into the Methods section) was applied to standardize sequencing depth prior to alpha and beta diversity analyses. We acknowledge that alternative normalization strategies, such as variance stabilization implemented in DESeq2 or compositional approaches (e.g., CLR transformation or ANCOM-based methods), have been proposed, particularly for differential abundance testing. However, previous studies have shown that rarefaction performs adequately for alpha and beta diversity analyses because it equalizes sampling effort across samples and facilitates comparison of ecological diversity metrics. In contrast, model-based or compositional approaches are generally better suited for differential abundance testing (https://doi.org/10.1186/s40168-017-0237-y, https://doi.org/10.1128/msphere.00354-23). We may argue that all methods have their limitations. DESeq2 for examples adopts assumptions from RNA-seq analyses, assuming that most features are equally abundant across samples. For microbiome data, this implies similar absolute abundances for most taxa, which may lack biological plausibility in many cases (https://doi.org/10.1111/1755-0998.13730). As our study did not include differential abundance testing, we consider rarefaction to be an appropriate approach for the analyses conducted. Nevertheless, we acknowledge that rarefaction has limitations, which are now discussed in the limitations section of the Discussion (L506-519). We hope the reviewer agrees to this.

1.4. Disk diffusion with 20 µg/ml fixed concentration, No CLSI breakpoints, No MIC determination limit clinical relevance.

1.4. Response: Our study aimed to explore the ecological diversity of antibiotic tolerance among environmental isolates associated with traditional dried plant foods, rather than to determine clinically defined resistance profiles. Therefore, disk diffusion assays using a fixed antibiotic concentration (20 µg/ml) were applied as a screening approach to identify isolates capable of growth under relatively high antibiotic exposure, similar to approaches used in environmental resistome studies (e.g., D’Costa et al., 2006). We acknowledge that the use of a fixed antibiotic concentration without determination of minimum inhibitory concentrations (MICs) and without applying CLSI clinical breakpoints does not allow classification of isolates as susceptible, intermediate, or resistant according to clinical standards. The assays therefore indicate phenotypic growth in the presence of antibiotics under the tested conditions, rather than clinically defined antimicrobial resistance. This clarification has now been added to the Methods (L 290-294) and the Discussion (L499-505) section of the revised manuscript.

1.5. Minor but frequent typographical inconsistencies, taxonomic formatting issues, some long sentences need tightening

1.5. Response: We closely checked the wording, eliminated issues und rephased sentences.

1.6. Need to avoid definitive species claims. Focus on vendor hygiene impact, handling practices and need for structured drying protocols

1.6. Response: The manuscript has been revised accordingly. Statements implying species-level identification were moderated throughout the manuscript to reflect the limitations of 16S rRNA gene sequencing. (Please also see our response to comment 1.2). In addition, the Discussion section was expanded to place greater emphasis on vendor-related factors, including hygiene practices, handling procedures, and drying conditions, which may influence the microbial composition of the dried plant products. We appreciate this suggestion, as it helped to strengthen the interpretation and clarity of the manuscript.

Reviewer: 2

Comments to the Author

General comments

The manuscript “Microbial diversity and health implications of traditional dried plant foods from South African informal markets” aims to assess the potential health implications associated to the microbiota of five traditional dried plant products (baobab, masau, nyii, dinawa and lude) sourced from three informal markets in South Africa, using 16S rRNA gene sequencing, quantitative real-time PCR, and functional assays. Although the study presents results that may be of interest to the field, some points require clarification and review before the manuscript can be considered for publication.

Response: We thank the reviewer for the careful evaluation of our manuscript and for recognizing the potential relevance of this study. We appreciate the constructive feedback provided and have revised the manuscript accordingly to address the points raised.

2.1. The results indicate that plant type is a determinant of community structure; however, this finding is presented mainly in a descriptive manner. I suggest expanding the discussion to include possible underlying mechanisms, such as differences in plant surface structure, chemical composition of the plant matrix, and other selective factors.

2.1. Response: Thank you for your comment. We agree and added more information about the drivers of the plant microbiome in the Discussion (L 470-485)

2.2. The limitations section is present but could be more comprehensive. The authors are encouraged to further discuss methodological and analytical constraints.

2.2. Response: The limitations section has been substantially expanded. Specifically, we now address (i) the limited number of vendors per product, (ii) the lack of detailed metadata on drying conditions, storage practices, and hygiene procedures, (iii) the limited taxonomic resolution and lack of functional inference associated with 16S rRNA gene sequencing, (iv) the relatively small number of cultured isolates analyzed compared to the total microbial diversity detected by sequencing, (v) the exclusive focus on bacterial communities, and (vi) the normalization of sequencing depth using rarefaction rather than alternative normalization strategies. In the section before we also highlighted the limitations of the antimicrobial resistance screening.

2.3. In several instances, statistical significance is reported without clearly indicating which group presented higher or lower values. The direction of the observed effects should be explicitly stated in the text.

2.3. Response: thank you for pointing this out. We closely checked the text for this issue.

2.4. Please, ensure a consistent format for figure citations throughout the manuscript (e.g., Figure 3A instead of Figure 3, A).

2.4. Response: we have thoroughly revised the text and format.

2.5. Statements regarding potential health benefits should be presented more cautiously and framed as hypotheses rather than implications supported by the current data.

2.5. Response: Thank you for this comment. We have thoroughly checked the text and changed all parts that overinterpreted the data. All species level-assignments from the amplicon dataset were deleted from the manuscript, focusing now only on genus level. Statements implying the presence of specific pathogenic species were replaced with more cautious wording. These revisions were implemented in the Abstract, Materials and Methods, Results, Discussion, and Conclusion. In the Methods section, we clarified that 16S rRNA gene sequencing does not provide information on functional traits or pathogenicity, and that the categorization of taxa as potentially beneficial or pathogenic is based on literature where these genera are commonly associated with such characteristics. We added that such assignments are probabilistic and not definitive at the species level (L222 - L229). The Results sections were strongly revised (L341-367), including the header and the corresponding table. All sentences suggesting functional potential were removed. In the Discussion we explicitly acknowledge the limitations of 16S rRNA sequencing and emphasized that conclusions regarding pathogenicity or virulence would require higher-resolution approaches such as whole-genome sequencing or targeted molecular assays (L464-469). We also revised the title of the manuscript as well as the featured image, which now focuses on the ecological importance, the impact of the vendor and food safety, instead for overclaiming health effects.

2.6. A general revision of the English language is required, as the manuscript contains typographical and grammatical errors.

2.6. Response: The manuscript has been carefully revised to improve clarity, grammar, and overall language quality. Typographical and grammatical errors were corrected throughout the text.

Specific comments

#1. L118: “…interchangeably when referring to dinawa and lude” → “…interchangeably when referring to dinawa and lude, respectively”

Response: thank you, it was revised.

#2. L199: “Thermo Fischer Scientific” → “Thermo Fisher Scientific”

Response: thank you, it was revised.

#3. L239: Include reference to the SILVA classification tool. The same applies to L259.

Response: thank you, it was revised.

#4. L314: At its first occurrence in the text, the acronym GCN should be written in full.

Response: The term gene copy number (GCN) is introduced in full at its first occurrence in the Methods section. Therefore, the acronym is used in abbreviated form in the Results section.

#5. L315: 3.99 x 109?? Please specify the unit.

Response: thank you, it was revised.

#6. L390-401: Please cite the figure where these results can be observed.

Response: thank you, it was revised.

#7. L426: Wicaksono et al. (Wicaksono et al., 2022) → Wicaksono et al. (2022). Please check the entire manuscript to ensure that references follow the journal style.

Response: thank you, we checked the manuscript thoroughly.

#8. L542: “and and assessing their potential transmission via plant-based foods” → “and assessing their potential transmission via plant-based foods”

Response: thank you, this part was strongly revised.

#9. Figure 2: The description of Panels G-H is missing.

Response: thank you, it was added.

#10. Figure 3: I suggest separating panels 3G–H and presenting them as a new figure to improve readability.

Response: We acknowledge this this suggestion. However, we believe that keeping them together helps preserve the logical flow and facilitates interpretation of the results. In its current layout, the figure fits well within an A4 page and will be provided in high resolution to ensure readability. For these reasons, we would prefer to retain the figure in its current form, unless the reviewer considers separation necessary.

#11. English language and typographical errors:

Examples include:

Paneals → Panels

Panals → Panels

eveneness → evenness

barcharts depicts → bar charts depict

Kruskal Wallis test →Kruskal–Wallis test

Response: We apologize for all the errors. We thoroughly checked English language and eliminated typographical errors.

Please perform a thorough language revision throughout the manuscript.

Response: a thorough language revision was performed. Thank you.

Reviewer: 3

Comments to the Author

Title

3.1. The title is clear and appropriate for the scope of the journal. However, the expression “health implications” may overstate the strength of the evidence presented, as the study is largely descriptive and based on 16S rRNA gene sequencing and in vitro assays. The wording should be softened to avoid implying direct health effects.

3.1. Response: Thank you for your comments. We carefully considered all suggestions and revised the manuscript accordingly to improve clarity, methodological description, and the interpretation of the results. We agree and changed the title to “Diversity and selected functional traits of microbiota associated with traditional dried plant foods from South African informal markets”. With the functional traits we refer to the cultivation-dependent tests. We hope the reviewer agrees.

Abstract

3.2. The abstract overinterprets associative findings. Correlations between richness and certain genera are framed as health-relevant implications without sufficient functional validation. The identification of Acinetobacter baumannii and Stenotrophomonas maltophilia as core microbiome members should be contextualized more cautiously, given the taxonomic resolution limits of 16S rRNA sequencing. The sample size and total number of samples analyzed should be clearly stated. Statements suggesting probiotic potential should be moderated.

3.2. Response: Thank you for your comment. The abstract has been revised strongly to avoid overinterpretation of associative findings and to moderate statements regarding potential health relevance and probiotic traits.

Introduction

3.3. The research question is not explicitly formulated and should be more clearly defined. The conceptual framing of “beneficial” versus “pathogenic” taxa is introduced without sufficient explanation, which later affects the interpretation of results. A clearer distinction between ecological description and health inference is needed. Furthermore, this section could contextualize the products that will be evaluated. Some information is included in the materials and methods section, but for the reader, it is better to be here.

3.3. Response: Thank you for this constructive suggestion. The Introduction has been revised to more clearly define the research objectives and to explicitly distinguish between ecological characterization of microbial communities and potential implications for food safety or health. We expanded the contextual description of the investigated plant products in the Introduction to improve readability and provide background information for the reader. The study aims are now explicitly stated at the end of the Introduction.

Materials and Methods

3.4. The decision to rarefy the dataset should be better justified, considering current debates on rarefaction versus alternative normalization approaches. The criteria used to define genera as “beneficial” or “potential human pathogens” are overly simplistic and require stronger justification or rephrasing. The lack of viability assessment significantly limits the interpretation of absolute abundance data, and this limitation should be emphasized earlier and more clearly. The limited number of vendors for certain products weakens statistical power and should be addressed more transparently.

3.4. Response: Thank you for your important comments.

Regarding the choice to rarefy our dataset: We agree about the importance of this point. In this study, rarefaction to the lowest read count (28,388 reads – now also included into the Methods section) was applied to standardize sequencing depth prior to alpha and beta diversity analyses. We acknowledge that alternative normalization strategies, such as variance stabilization implemented in DESeq2 or compositional approaches (e.g., CLR transformation or ANCOM-based methods), have been proposed, particularly for differential abundance testing. However, previous studies have shown that rarefaction performs adequately for alpha and beta diversity analyses because it equalizes sampling effort across samples and facilitates comparison of ecological diversity metrics. In contrast, model-based or compositional approaches are generally better suited for differential abundance testing (https://doi.org/10.1186/s40168-017-0237-y, https://doi.org/10.1128/msphere.00354-23). We may argue that all methods have their limitations. DESeq2 for examples adopts assumptions from RNA-seq analyses, assuming that most features are equally abundant across samples. For microbiome data, this implies similar absolute abundances for most taxa, which may lack biological plausibility in many cases (https://doi.org/10.1111/1755-0998.13730). As our study did not include differential abundance testing, we consider rarefaction to be an appropriate approach for the analyses conducted. Nevertheless, we acknowledge that rarefaction has limitations, which are now discussed in the limitations section of the Discussion (L506-519). We hope the reviewer agrees to this.

We further agree that “beneficial” and “potentially pathogenic” are highly simplistic. We have revised the entire manuscript changing all parts that overinterpreted the data. All species level-assignments from the amplicon dataset were deleted from the manuscript, focusing now only on genus level. Statements implying the presence of specific pathogenic species were replaced with more cautious wording. In the Methods section, we clarified that 16S rRNA gene sequencing does not provide information on functional traits or pathogenicity, and that the categorization of taxa as potentially beneficial or pathogenic is based on literature where these genera are commonly associated with such characteristics. We added that such assignments are probabilistic and not definitive at the species level (L222 - L229). The Results sections were strongly revised (L341-367), including the header and the corresponding table. All sentences suggesting functional potential were removed. In the Discussion we explicitly acknowledge the limitations of 16S rRNA sequencing and emphasized that conclusions regarding pathogenicity or virulence would require higher-resolution approaches such as whole-genome sequencing or targeted molecular assays (L464-469). We also revised the title of the manuscript as well as the featured image, which now focuses on the ecological importance, the impact of the vendor and food safety, instead for overclaiming health effects.

Overall, we believe that we did our best not to overinterpret the data, but at the same time, we also believe that a very careful discussion that Lactobacillus is rather considered as a potential beneficial bacterium and that Acinetobacter and Stenotrophomonas are often and rather considered to have an opportunistic pathogenic potential, is still appropriate, also from a taxonomic and very descriptive level.

We added the limitations of the qPCR in the Methods (L171-172) and Discussion (L429-432).

We added the limitations about low number of vendors for certain products in the Methods (L127-129), the Discussion (L411-412) and the limitations section (starting at L506).

Results

3.5. Several interpretations go beyond what the data support. Correlations between richness and “beneficial taxa” are presented in a way that suggests functional consequences, which cannot be inferred from 16S data alone.

3.5. Response: Thank you for your comment. We have strongly revised all parts that claim health effects. Please also see our response to your previous comment.

3.6. The concept of a “core microbiome” dominated by potential pathogens requires more careful discussion, especially given species-level uncertainty.

3.6. Response: We revised this results section significantly, as well as the related text in the discussion.

3.7. The functional assays were conducted on a limited number of isolates relative to total ASVs, and this limitation should be clearly acknowledged when concluding functional potential. The antimicrobial resistance results are important but should avoid implying direct public health risk without genetic confirmation of resistance determinants.

3.7. Response: We acknowledged the limitations of the low number of isolates relative to total ASVs in the Results (L399-401) and the Discussion (L420-422), and in the limitations section starting at L506). Thank you for pointing this out.

Regarding the antimicrobial resistance tests: We fully agree that no direct health risk can be implied by the antimicrobial resistance tests. We revised Abstract, Results, and Discussion. Our study aimed to explore the ecological diversity of antibiotic tolerance among environmental isolates associated with traditional dried plant foods, rather than to determine clinically defined resistance profiles. Therefore, the disk diffusion assays using a fixed antibiotic concentration (20 µg/ml) were applied as a screening approach to identify isolates capable of growth under relatively high antibiotic exposure, similar to approaches used in environmental resistome studies (e.g., D’Costa et al., 2006). In the Discussion we now acknowledge that the use of a fixed antibiotic concentration without determination of minimum inhibitory concentrations (MICs) and without applying CLSI clinical breakpoints does not allow classification of isolates as resistant according to clinical standards. The assays therefore indicate phenotypic growth in the presence of antibiotics under the tested conditions, rather than clinically defined antimicrobial resistance. This clarification has now been added to the Methods (L 290-293) and the Discussion (L499-505) section of the revised manuscript.

Discussion

3.8. The discussion contains speculative elements that extend beyond the presented evidence. The dichotomous framing of taxa as beneficial versus pathogenic oversimplifies microbial ecology and should be revised.

Statements implying possible modulation of the human gut microbiome by these foods are hypothetical and should be clearly labeled as such.

The interpretation of antimicrobial resistance findings would benefit from a more cautious tone, emphasizing that phenotypic resistance in environmental isolates does not necessarily equate to transmission risk. Some arguments rely on comparisons with fermented foods or industrial systems that are not directly tested in this study and should be reframed as perspectives rather than conclusions.

3.8. Response: we thank you for the suggestions. The Discussion has been revised to ensure that interpretations remain closely aligned with the presented data. The dichotomous framing of taxa as “beneficial” versus “pathogenic” has been removed and replaced with more cautious wording indicating that these genera are reported in the literature to include beneficial or clinically relevant species, while acknowledging the limitations of genus-level identification based on 16S rRNA sequencing. Statements regarding potential modulation of the human gut microbiome have been revised to clearly indicate that such effects remain hypothetical (L464-469).

The interpretation of antimicrobial resistance results has also been moderated. We now emphasize that the assays represent phenotypic growth in the presence of antibiotics and do not necessarily indicate the presence of transferable resistance genes or a direct transmission risk to humans. Please see also our response to your previous comment. In addition, the discussion was expanded to better address vendor-related factors, including hygiene practices, handling conditions, and drying procedures, and to highlight the potential value of structured drying protocols.

Finally, the section discussing plant-type effects has been expanded to include possible ecological mechanisms (e.g., differences in plant surface structure and chemical composition), and the limitations section has been strengthened to further address methodological constraints of the study.

Conclusion

3.9. The conclusion overstates the health-related implications of the findings. It should focus more strictly on the ecological characterization of microbiota and the need for improved hygiene practices, rather than implying potential health benefits or risks that were not directly measured. The wording should reflect the exploratory and descriptive nature of the study.

3.9. Response: Thank you for your suggestions. The conclusion has been revised to better reflect the exploratory and descriptive nature of the study. Health-related interpretations were removed or substantially moderated, and the focus is now placed on the ecological characterization of the microbiota associated with traditional dried plant foods. The revised conclusion emphasizes the role of plant species identity and vendor-related factors in shaping microbial community composition, as well as the importance of hygienic handling, storage, and drying practices in informal market settings. In addition, references to potential health benefits or risks that were not directly assessed have been removed or rephrased to avoid overinterpretation.

**Reviews of manuscript FEMSMC-2026-008.R1**

**Reviewer 2**

**Recommendation: Accept**

The authors addressed the comments carefully and thoroughly. I am satisfied with the revisions presented, which significantly improved the overall quality, clarity, and scientific rigor of the manuscript. The revised version demonstrates better organization, and a more robust discussion of the results. The authors' responses to the comments were appropriate and well-founded, and the modifications made throughout the text effectively strengthen the manuscript. Overall, the manuscript is now significantly improved compared to the original version, and the revisions have addressed the main concerns raised in the previous review.

**Reviewer 3**

**Recommendation: Major Revision**

The authors have made a clear effort to revise the manuscript and address several of the concerns raised in the previous round. In particular, improvements are noted in reducing overinterpretation of 16S rRNA gene sequencing data, removing species-level assignments, and including a more explicit discussion of methodological limitations. These changes represent an important step toward improving the scientific rigor of the manuscript. However, despite these improvements, relevant issues remain and must be addressed before the manuscript can be considered for publication.

Abstract

The abstract requires further revision to avoid residual overinterpretation. Although the authors have moderated some statements, there are still implications regarding potential health relevance and functional roles of the detected microbiota that are not directly supported by the data. The abstract should be strictly aligned with the scope of 16S rRNA gene sequencing and avoid any suggestion of confirmed functional or pathogenic roles. All statements related to health implications should be clearly framed as hypotheses or removed if not directly supported.

Introduction

The introduction has been improved but still remains largely descriptive. The authors should strengthen the scientific framing by more clearly defining the knowledge gap and the specific contribution of this study. In addition, the rationale linking microbial diversity in traditional dried plant foods to health implications should be presented more cautiously, avoiding any implicit causal assumptions. A more critical and focused narrative rather than a broad descriptive overview is needed.

Materials and Methods

The methodological section is generally acceptable; however, the limitations associated with the analytical approaches should be more clearly acknowledged. In particular, the use of rarefaction, although justified, should be discussed more critically in light of current practices in microbiome data analysis. Additionally, the interpretation of antibiotic tolerance assays should be carefully contextualized as non-clinical screening, and this limitation should be consistently reflected throughout the manuscript.

Results

The results are clearly presented, but they remain predominantly descriptive. The authors should improve the clarity of their reporting by explicitly indicating the direction of statistically significant differences when they are mentioned. Furthermore, the connections among the datasets presented in the study could be strengthened to provide a more coherent narrative.

Discussion

The discussion has been expanded but still lacks sufficient analytical depth. While the authors have reduced overstatements, there are still instances where taxonomic findings are implicitly linked to functional or health-related implications. These associations should be further refined and clearly presented as speculative when not directly supported by the data. The discussion should also provide a more critical interpretation of the findings, particularly regarding the ecological drivers of microbial diversity and their relevance to food safety and handling practices. A clearer distinction between observed results and literature-based assumptions is still needed.

**Associate Editor Recommendation: Major Revision**

**EIC Decision: Major Revision**

**Author's Response to Decision Letter for (FEMSMC-2026-008.R1)**

Reviewers' Comments to Author/s:

Reviewer: 2

Comments to the Author

The authors addressed the comments carefully and thoroughly. I am satisfied with the revisions presented, which significantly improved the overall quality, clarity, and scientific rigor of the manuscript. The revised version demonstrates better organization, and a more robust discussion of the results. The authors' responses to the comments were appropriate and well-founded, and the modifications made throughout the text effectively strengthen the manuscript. Overall, the manuscript is now significantly improved compared to the original version, and the revisions have addressed the main concerns raised in the previous review.

Response: We sincerely thank the reviewer for the positive evaluation of our revised manuscript and for acknowledging the improvements made. We appreciate the reviewer’s constructive feedback throughout the review process, which helped strengthen the clarity and scientific rigor of the manuscript.

Reviewer: 3

Comments to the Author

The authors have made a clear effort to revise the manuscript and address several of the concerns raised in the previous round. In particular, improvements are noted in reducing overinterpretation of 16S rRNA gene sequencing data, removing species-level assignments, and including a more explicit discussion of methodological limitations. These changes represent an important step toward improving the scientific rigor of the manuscript. However, despite these improvements, relevant issues remain and must be addressed before the manuscript can be considered for publication.

Response: We thank the reviewer for recognizing the efforts made to revise the manuscript and for acknowledging the improvements. We appreciate the reviewer’s continued careful evaluation and have addressed the remaining concerns in detail below.

Abstract

The abstract requires further revision to avoid residual overinterpretation. Although the authors have moderated some statements, there are still implications regarding potential health relevance and functional roles of the detected microbiota that are not directly supported by the data. The abstract should be strictly aligned with the scope of 16S rRNA gene sequencing and avoid any suggestion of confirmed functional or pathogenic roles. All statements related to health implications should be clearly framed as hypotheses or removed if not directly supported.

Response: The abstract has been revised to further reduce overinterpretation and to ensure strict alignment with the scope of 16S rRNA gene sequencing data. We deleted all functional interpretations and added the sentence: “Notably, health implications of detected taxa cannot be inferred from genus-level identification based on 16S rRNA gene sequencing.” alongside further revisions to clarify the methods used. We hope the reviewer agrees to this revision.

Introduction

The introduction has been improved but still remains largely descriptive. The authors should strengthen the scientific framing by more clearly defining the knowledge gap and the specific contribution of this study. In addition, the rationale linking microbial diversity in traditional dried plant foods to health implications should be presented more cautiously, avoiding any implicit causal assumptions. A more critical and focused narrative rather than a broad descriptive overview is needed.

Response: The Introduction has been revised to strengthen the scientific framing by more clearly defining the knowledge gap regarding the microbial characterization of traditional dried plant foods sold in informal markets (L 92-98). Descriptive background information has been reduced and condensed, particularly in the section describing the food-gut axis, and the investigated plant species. However, for the latter we retained essential contextual information requested during the previous revision round by another reviewer. In addition, statements linking microbial diversity to potential health implications have been moderated and revised to explicitly acknowledge methodological limitations (L71-77). Finally, the study objectives were refined to more clearly highlight the specific contribution of this work as providing baseline data on microbial community composition and selected phenotypic traits associated with traditional dried plant foods sold in informal markets (L 124-131).

Materials and Methods

The methodological section is generally acceptable; however, the limitations associated with the analytical approaches should be more clearly acknowledged. In particular, the use of rarefaction, although justified, should be discussed more critically in light of current practices in microbiome data analysis. Additionally, the interpretation of antibiotic tolerance assays should be carefully contextualized as non-clinical screening, and this limitation should be consistently reflected throughout the manuscript.

Response: The limitations associated with rarefaction have been clarified in the Methods section (L229-233). Additionally, the limitations paragraph in the Discussion has been largely expanded to more critically discuss the use of rarefaction in light of current microbiome data analysis practices and to reference alternative normalization strategies (L658-665). The limitations of the method used to assess antimicrobial phenotypes were further expanded in the Results section (“These results indicate phenotypic tolerance under the tested conditions, which included a fixed antibiotic concentration (20 µg/ml) and represent a method applied in environmental resistome studies to detect strong resistance phenotypes (D’Costa et al., 2006; Walsh & Duffy, 2013). The presence of specific antimicrobial resistance genes or clinically relevant resistance mechanisms was not assessed in this study.”). We believe that, by stating this limitation in the Methods (“Disk diffusion assays using a fixed antibiotic concentration (20 µg/ml) were applied as a screening approach to identify isolates capable of growth under relatively high antibiotic exposure. Similar screening strategies using elevated antibiotic concentrations have previously been applied in environmental resistome studies to detect strong resistance phenotypes (D’Costa et al., 2006; Walsh & Duffy, 2013; Wicaksono et al., 2023). … The purpose of this assay was to provide a comparative overview of antibiotic tolerance within the environmental isolate collection rather than to classify isolates according to clinical susceptibility standards.”), and in the Discussion (“Importantly, assays were conducted at a fixed antibiotic concentration (D’Costa et al., 2006) without determination of minimal inhibitory concentration or CLSI breakpoints, thus, the results should be interpreted as phenotypic tolerance under the tested conditions rather than clinically defined resistance. Moreover, phenotypic resistance observed in environmental isolates does not necessarily indicate the presence of transferable antimicrobial resistance genes or imply a direct transmission risk to humans. Confirming such risks would require genomic analyses targeting specific resistance determinants and mobile genetic elements.”), the limitations of the method are now described sufficiently critically, and we hope the reviewer agrees with this clarification.

Results

The results are clearly presented, but they remain predominantly descriptive. The authors should improve the clarity of their reporting by explicitly indicating the direction of statistically significant differences when they are mentioned.

Furthermore, the connections among the datasets presented in the study could be strengthened to provide a more coherent narrative.

Response: The Results section has been revised to improve clarity by explicitly indicating the direction of statistically significant differences (especially L310-324). Following this description, we also added an additional observation “Vendor-specific effects were product-dependent, and no consistent pattern was observed indicating that a particular vendor was systematically associated with higher or lower bacterial abundance or diversity across products.” This was also added to the Discussion (L437-439). We thank the reviewer for pointing that out with the comment. In addition, short summary and transition sentences have been added at the end of each Results subsection to better connect the individual datasets and provide a more coherent narrative flow throughout the Results section. We hope these revisions address the reviewer’s expectations.

Discussion

The discussion has been expanded but still lacks sufficient analytical depth. While the authors have reduced overstatements, there are still instances where taxonomic findings are implicitly linked to functional or health-related implications. These associations should be further refined and clearly presented as speculative when not directly supported by the data. The discussion should also provide a more critical interpretation of the findings, particularly regarding the ecological drivers of microbial diversity and their relevance to food safety and handling practices. A clearer distinction between observed results and literature-based assumptions is still needed.

Response: Thank you for your comment. First, we acknowledge that some parts of the Discussion required a clearer distinction between observed results and literature-based assumptions. We have revised all relevant sections and, regarding functional interpretation, explicitly added a clarification stating that interpretations informed by previous literature are intended to provide contextual support rather than to imply direct functional inference from the present dataset, as this study is based solely on genus-level taxonomic data and does not allow functional or health-related conclusions (L586–589). In addition, all statements linking taxonomic findings to functional or health-related implications were carefully revised to emphasize their speculative nature or were removed entirely (e.g., from the first paragraph of the Discussion and from the Conclusion). We also provided additional ecological context, particularly in the revised Conclusion. We hope that these revisions strengthen the analytical depth of the Discussion and improve the clarity of the manuscript.

**Reviews of manuscript FEMSMC-2026-008.R2**

**Reviewer 3**

**Recommendation: Accept**

The authors have adequately addressed the main concerns raised in the previous round of review. The revised manuscript shows clear improvements, particularly in reducing overinterpretation of 16S rRNA data, strengthening the discussion of methodological limitations, and improving overall clarity and structure. The abstract, introduction, and discussion are now more cautious and appropriately aligned with the scope of the data, and the limitations are more consistently acknowledged throughout the manuscript. Although some sections remain slightly descriptive, this does not compromise the overall quality at this stage. Therefore, the manuscript is suitable for acceptance.

**Associate Editor Recommendation: Accept**

**EIC Decision: Accept**
